# Discovery of microRNA-like Small RNAs in Pathogenic Plant Fungus *Verticillium nonalfalfae* Using High-Throughput Sequencing and qPCR and RLM-RACE Validation

**DOI:** 10.3390/ijms23020900

**Published:** 2022-01-14

**Authors:** Taja Jeseničnik, Nataša Štajner, Sebastjan Radišek, Ajay Kumar Mishra, Katarina Košmelj, Urban Kunej, Jernej Jakše

**Affiliations:** 1 Department of Agronomy, Biotechnical Faculty, University of Ljubljana, 1000 Ljubljana, Slovenia; taja.jesenicnik@bf.uni-lj.si (T.J.); natasa.stajner@bf.uni-lj.si (N.Š.); katarina.kosmelj@bf.uni-lj.si (K.K.); urban.kunej@bf.uni-lj.si (U.K.); 2 Plant Protection Department, Slovenian Institute of Hop Research and Brewing, 3310 Žalec, Slovenia; sebastjan.radisek@ihps.si; 3 Biology Centre of the Czech Academy of Sciences, Department of Molecular Genetics, Institute of Plant Molecular Biology, Branišovská 31, 37005 České Budějovice, Czech Republic; ajaymishra24@umbr.cas.cz

**Keywords:** RNA interference, microRNA-like RNAs, plant-pathogen interactions, fungal pathogen, *Verticillium nonalfalfae*

## Abstract

*Verticillium nonalfalfae* (*V. nonalfalfae*) is one of the most problematic hop (*Humulus lupulus* L.) pathogens, as the highly virulent fungal pathotypes cause severe annual yield losses due to infections of entire hop fields. In recent years, the RNA interference (RNAi) mechanism has become one of the main areas of focus in plant—fungal pathogen interaction studies and has been implicated as one of the major contributors to fungal pathogenicity. MicroRNA-like RNAs (milRNAs) have been identified in several important plant pathogenic fungi; however, to date, no milRNA has been reported in the *V. nonalfalfae* species. In the present study, using a high-throughput sequencing approach and extensive bioinformatics analysis, a total of 156 milRNA precursors were identified in the annotated *V. nonalfalfae* genome, and 27 of these milRNA precursors were selected as true milRNA candidates, with appropriate microRNA hairpin secondary structures. The stem-loop RT-qPCR assay was used for milRNA validation; a total of nine *V. nonalfalfae* milRNAs were detected, and their expression was confirmed. The milRNA expression patterns, determined by the absolute quantification approach, imply that milRNAs play an important role in the pathogenicity of highly virulent *V. nonalfalfae* pathotypes. Computational analysis predicted milRNA targets in the *V. nonalfalfae* genome and in the host hop transcriptome, and the activity of milRNA-mediated RNAi target cleavage was subsequently confirmed for two selected endogenous fungal target gene models using the 5′ RLM-RACE approach.

## 1. Introduction

The soil fungus *Verticillium nonalfalfae* (*V. nonalfalfae*) is a causal agent of vascular wilt diseases in many important agriculture crops, trees and other woody plants [1,2]. Taxonomically, *V. nonalfalfae* belongs to a group of 10 species that form the anamorphic genus *Verticillium sensu* stricto in the class Sordariomycetes in the phylum Ascomycota (www.mycobank.org, accessed on 21 September 2021). The fungus is able to survive in soil with its melanised resting mycelia, which germinate in response to root exudates. Penetration hyphae infect host roots, and the infection progresses with the colonization of xylem vessels and results in the wilting of the affected parts of plants or, in the case of severe disease forms, dieback of the entire plant [3]. Hop (*Humulus lupulus* L.) is one of the most susceptible hosts to *V. nonalfalfae*, as the fungus causes outbreaks in many hop growing regions of Europe and worldwide [4,5]. Highly virulent pathotypes cause the most severe outbreaks in hops, resulting in rapid plant dieback, which is known as lethal wilt. Lethal wilt rapidly progresses throughout entire hop fields, leads to extensive yield losses and affects hop production for several years. On the other hand, less virulent *V. nonalfalfae* pathotypes have been identified, which affect only lower parts of plants or individual bines and rarely cause plant dieback [6,7]. To date, no specific treatment of affected plants with *V. nonalfalfae* infection has been developed, and the resting mycelia enable the fungus to be preserved in soil for many years [8]. Disease management thus relies only on breeding and growing resistant hop cultivars, soil sanitation methods and ensuring appropriate phytosanitary measures [9].

In recent years, there has been renewed and growing interest in the RNA interference (RNAi)-mediated regulatory mechanism. The core components of RNAi have been identified in several fungal species. The genome annotation [10] of *V.nonalfalfae* led to the identification of two RNA-dependent RNA polymerases (RdRP), two Dicer-like (DCL) enzymes and two Argonaut (AGO) proteins as well as characterization of their conserved RNAi domains [11]. It has been demonstrated that the core RNAi genes of *V. nonalfalfae* are differentially expressed in different *V. nonalfalfae* pathotypes and in different fungal tissues, suggesting a possible role for RNAi in the pathogenicity of the fungus [11]. The silencing of specific genes in the conserved RNAi pathway is mediated by 21- to 25-nt long small RNA (sRNA) molecules, complementary to their target messenger RNA (mRNA) transcripts [12]. Eukaryotic sRNAs are generally divided into three groups, of which microRNAs (miRNAs), characterized by the typical hairpin secondary structure of the miRNA precursor, where the mature miRNA resides on one strand of the hairpin structure [13,14,15], are the class of sRNAs that have been most extensively studied in the fungal kingdom. MiRNAs are a group of RNA molecules encoded by miRNA genes distributed throughout the entire genome; most miRNA genes are noncoding, and some miRNAs reside within intronic regions, untranslated regions and even exonic regions of protein coding genes [15]. In fungi, miRNA precursors are typically transcribed by RNA polymerase III and are processed only with Dicer enzymes to form mature miRNAs [16]; therefore, fungal miRNAs are referred to as microRNA-like RNAs (milRNAs). MilRNAs are important regulators of fungal gene expression and potentially contribute to fungal pathogenicity, as demonstrated by milRNA expression studies in different developmental stages, under different environmental conditions and at the level of host-pathogen interactions [16,17]. High-throughput sequencing coupled with a bioinformatics approach has opened the avenue to milRNA identification studies in several ascomycete and basidiomycete fungal species, including important pathogenic plant fungal species, such as *Sclerotinia sclerotiorum* [18], *Fusarium* spp. [19,20], *Puccinia* spp. [21,22] and *V. nonalfalfae* related *Verticillium dahliae* (*V. dahliae*) species [23,24].

Emerging research has suggested that pathogen-derived endogenous sRNAs can translocate from pathogens into host plant cells during infection to trigger gene silencing of host immunity, which describes an interesting mechanism of cross-kingdom RNAi during the host-pathogen interaction [25,26,27,28,29]. RNAi signals can successfully translocate in the plant vasculature protected in the form of extracellular vesicles, and thus travel long distances via phloem-based movement [27,28,30,31]. The fungal pathogen *Botrytis cinerea* (*B. cinerea*) is the first explicit example of this type of RNAi signal translocation, in which it has been shown that sRNA effectors derived from long terminal repeat (LTR)-retrotransposons can translocate into plant hosts (*Arabidopsis thaliana* and *Solanum lycopersicum*) and suppress their immunity genes by hijacking the AGO protein and the RNA-induced silencing complex (RISC) [32]. Remarkably, cross-kingdom RNAi has been shown to be bidirectional owing to the virtue of the translocation of plant endogenous sRNAs into fungi, as several host sRNAs have been detected in the soil-borne fungal pathogen *V. dahliae*, targeting the fungal genes involved in invasion and pathogenicity [32,33]. Subsequently, exploration of the cross-kingdom sRNA transmission and RNA silencing mechanism associated with targets residing in the host genome has opened new avenues to understand plant–microbe interactions and to further develop an intelligent strategy for controlling broad host range pathogens. As a best suited alternative to transgenic approaches, this new knowledge has been exploited to design the modern plant defense strategy termed spray-induced gene silencing (SIGS), with direct application of pathogen gene-targeting dsRNAs or sRNAs (environmental RNAi) onto the plant surface or post-harvest products, resulting in the suppression of pathogen virulence and conferring efficient disease control [34]. 

To our knowledge, this is the first comprehensive report on the identification of sRNAs, and specifically milRNAs, in *Verticillium nonalfalfae*. In the present study, we exploited a next generation sequencing (NGS)-based approach to identify sRNAs in two Slovenian pathotypes, the highly virulent T2 isolate and the less virulent Rec isolate of *V. nonalfalfae*. Exploiting omics approaches, we mined the milRNA precursors and the targets of mature milRNAs in the *V. nonalfalfae* genome. To this end, we performed experimental validation of several predicted candidate milRNAs and their expression patterns in different pathotypes. Moreover, the potential targets were experimentally validated to confirm the activity of milRNA-mediated RNAi in *V. nonalfalfae* fungi.

## 2. Results

### 2.1. Overview of Verticillium nonalfalfae sRNAs and NGS Sequencing

In total, eight small RNA NGS libraries were constructed, representing four different tissues or growth stages of highly virulent T2 and less virulent Rec *V. nonalfalfae* isolates. Small RNA sequencing performed using the NGS Ion Torrent platform generated over 40 million sequencing reads for each sequencing matrix (T2 and Rec). On average, for each of the eight sequenced samples (Rec_XSM mycelia, Rec_CD mycelia, Rec_conidia, Rec_resting mycelia, T2_XSM mycelia, T2_CD mycelia, T2_conidia and T2_resting mycelia), approximately 10 million reads were obtained via high-throughput sequencing (Table 1). The obtained raw data were submitted to the NCBI Sequence Read Archive under the accession number PRJNA624041. The adaptor and barcode trimmed clean sequence reads were subjected to QC analysis, revealing that the length of the clean reads ranged from 10 to 40 nt, with peaks at approximately 15 and 30 nt (Figure S1). Among the clean reads, approximately 18% were observed to be unique reads in each of the eight data sets. The alignment of clean reads against the Rfam database showed that 5% of the sequences aligned to rRNA, tRNA, snRNA and snoRNA (Figure S2) and were subsequently removed from the milRNA prediction analysis.

### 2.2. Identification of Potential milRNAs

**Figure 1:**
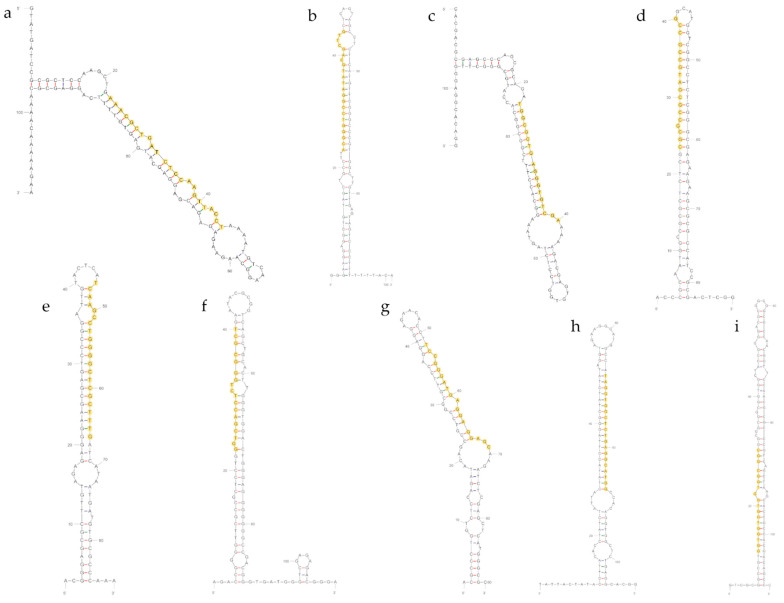
Prediction of the *V. nonalfalfae* precursor secondary structures; (**a**)—vna-miR-1, (**b**)—vna-miR-2, (**c**)—vna-miR-3, (**d**)—vna-miR-4, (**e**)—vna-miR-5, (**f**)—vna-miR-6, (**g**)—vna-miR-7, (**h**)—vna-miR-8 and (**i**)—conserved-miR-7044; the mature milRNA sequence is highlighted in yellow.

The MIReNA [35] algorithm predicted a total of 156 potential *V. nonalfalfae* milRNA precursors in all eight sRNA data sets, and among them, 103 were predicted from the four datasets of the less virulent Rec isolate and 53 were predicted in the four datasets of the highly virulent T2 isolate. All 156 predicted candidate precursors from all the datasets were folded into secondary structures using the mfold online tool, and the structures were manually inspected. The typical hairpin secondary structure (Figure 1) was confirmed for a total of 46 milRNA precursors. In the next step, using the criteria for plant miRNAs [36], all the precursors were categorized based on their secondary structure and AMFE (adjusted minimal free energy) and MFEI (minimal free energy index) values (normalization of minimal free energy (MFE) based on the precursor length and proportion of guanine and cytosine), which led to the identification of 27 true milRNA candidates (18 for the Rec isolate and 9 for the T2 isolate, respectively) (Table S1). The genomic origin of the precursors for all 27 true milRNA candidates was further examined by BLASTX analysis, revealing a total of 16 precursors residing within the coding regions of the *V. nonalfalfae* gene models. Conversely, 11 milRNA candidate precursors did not show any hits within coding regions of proteins. The selected 27 true milRNA candidates were further experimentally validated by qPCR.

### 2.3. Stem-Loop RT-qPCR Validation and Expression Analysis of Verticillium nonalfalfae milRNAs

The stem-loop RT-qPCR method [37] was optimized for small RNA amplification, and, then, used to monitor the expression profiles of the selected true milRNA candidates. All 27 milRNA candidates were successfully reverse transcribed into cDNA using designed specific stem-loop primers (Table S2). RT-qPCR amplification using the 27 designed milRNA-specific qPCR primers (Table S3) showed amplification of all 27 milRNAs in both the Rec and T2 isolates; however, the Ct (cycle threshold) values of the amplified products varied among the 27 selected milRNAs. With the selected approach, 15 milRNAs were amplified by the specific primers in the Rec and T2 pathotypes, and among them, eight milRNAs (vna-miR-1, vna-miR-2, vna-miR-3, vna-miR-4, vna-miR-5, vna-miR-6, vna-miR-7 and vna-miR-8) were amplified with primers covering the entire milRNA sequence or a one nucleotide shorter sequence, thus ensuring amplification of the specific milRNA sequence.

For the remaining seven milRNAs, whose specificity could not be confirmed using the primers used for amplification, the PCR products were cloned into plasmid vectors and sequenced with the Sanger-based approach [38]. The sequences were manually inspected, and the specific milRNA sequence was confirmed for one milRNA (conserved-miR-7044), whereas for the remaining six milRNAs, the obtained sequences contained two or more mismatches within the mature milRNA region that were not covered by the designed qPCR primers (Figure S3). Using the selected validation approach, a total of nine *V. nonalfalfae* milRNAs were experimentally confirmed, and they were named in accordance with the established miRNA nomenclature (Table 2). 

Eight *V. nonalfalfae* milRNAs were found to be novel without any conservations or hits in the miRBase database and were thus designated as “vna” for *V. nonalfalfae*. One milRNA was conserved in the database, with a hit for a mouse miRNA, mmu-miR-7044 (Figure S4). All nine milRNAs were present in the Rec and T2 isolates, as they were amplified from RNA derived from tissues of the two isolates. The lengths of the predicted and confirmed mature milRNAs ranged from 18 to 23 nt, and the mature milRNA resided on one hand of the hairpin precursor. All nine identified and confirmed milRNAs showed the typical milRNA secondary structure (Figure 1).

**Figure 2:**
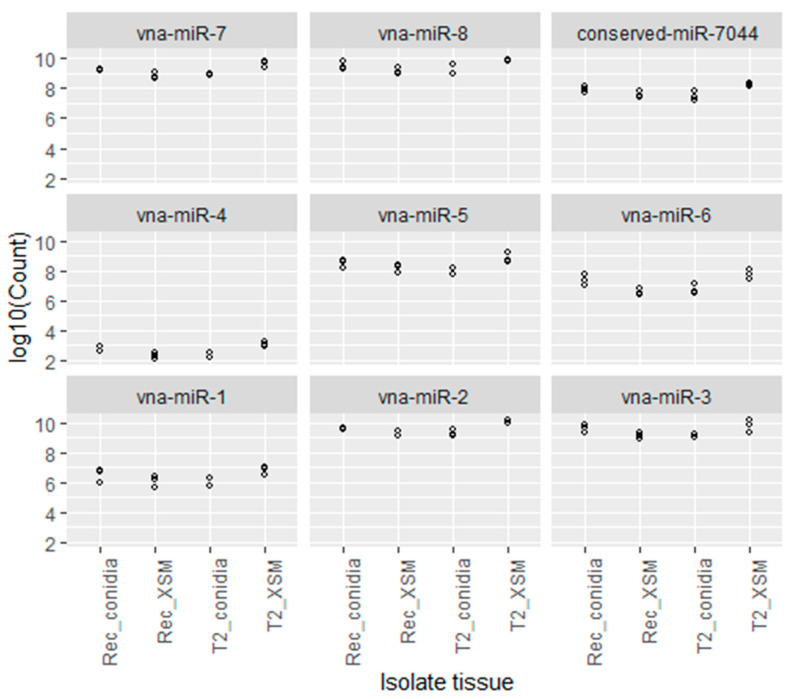
MilRNA copy number plotted on log10 scale for each confirmed *V. nonalfalfae* milRNA in each of the four investigated pathotype-tissue combinations; the use of 10 for the logarithmic base allows the power of the count values to be read directly from the plot. Statistically significant differences between the samples were tested using the one-way ANOVA methodology.

To gain insight into the potential effects of milRNAs on the RNAi-mediated growth, development and pathogenicity of *V. nonalfalfae*, we investigated the expression patterns of all nine validated milRNAs in two specific tissues, conidia and mycelia grown in XSM, of two different isolates (the highly virulent T2 and less virulent Rec isolates). The expression of milRNAs was quantified using the absolute quantification approach, and log10 of the milRNA copy number was plotted for all nine milRNAs in four investigated combinations: Rec_XSM mycelia, Rec_conidia, T2_XSM mycelia and T2_conidia (Figure 2).

All nine *V. nonalfalfae* milRNAs were found to be expressed in all the investigated tissues of the two pathotypes; however, the most abundantly expressed milRNAs were vna-miR-2, vna-miR-3, vna-miR-7 and vna-miR-8 because they had the highest milRNA log10 copy numbers (Figure 2). In the highly virulent pathotype T2, the statistically significant (*p* < 0.001) highest expression of all the milRNAs was detected in mycelia grown in XSM, in which the mean count of the milRNA copy number was approximately two-fold greater than the mean count for conidia (Figure S4). On the other hand, in the less virulent Rec isolate, all the milRNAs were most abundantly expressed in conidia (Figure 2). The comparison of the milRNA copy number between isolates revealed that their mean count is two-fold higher in the mycelia of the T2 isolate than in the Rec isolate, indicating that in the highly virulent pathotype the investigated milRNAs are more abundantly expressed (Figure S4). In the conidia, the milRNAs were more abundantly expressed in the Rec isolate than in the T2 isolate.

### 2.4. Target Prediction and GO Clustering Analysis

**Figure 3:**
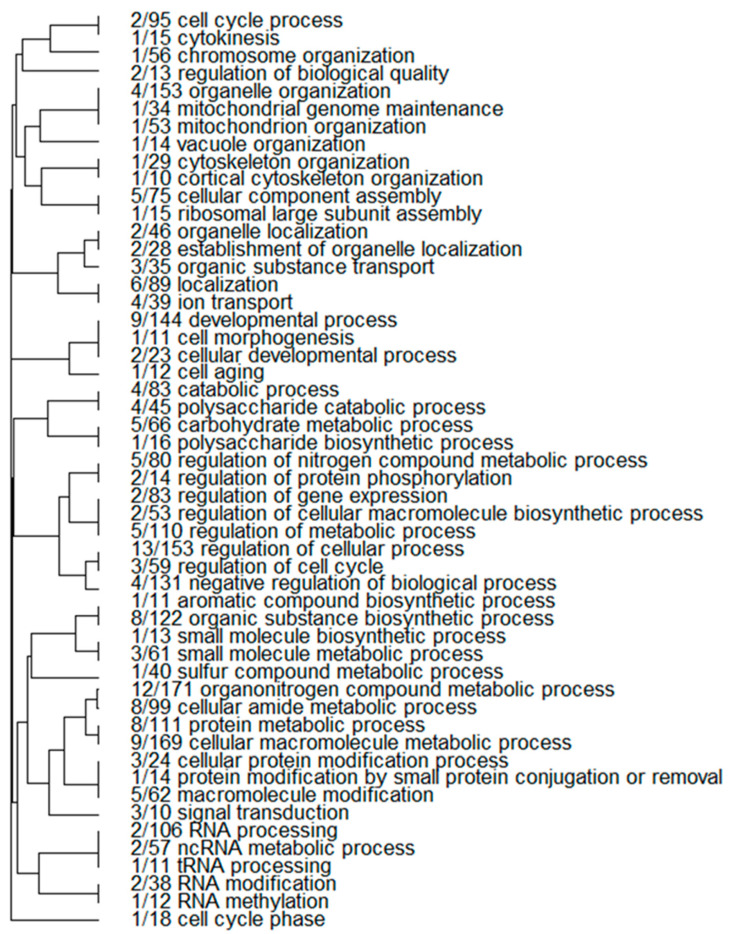
Predicted endogenous target clustering of the selected *V. nonalfalfae* gene models by their biological process according to enriched gene ontologies; the values represent the number of selected milRNA targets in each enriched GO annotation.

The targets of the experimentally validated milRNAs were predicted in the genome of *V. nonalfalfae* and in the transcriptome of its host hop plant. The list of all predicted targets were stored in the FigShare repository and are available with the URL: https://doi.org/10.6084/m9.figshare.11847177.v1 (accessed on 17 November 2021). In the fungal genome, a total of 307 genes were predicted to contain a target site for the nine validated milRNAs of *V. nonalfalfae*. Among them, 239 were found to be unique target sites, whereas 68 genes contained target sites for two or more milRNAs. A total of 194 and 78 target genes were predicted for conserved-miR-7044 and vna-miR-3, respectively, supporting the widely accepted notion that a single miRNA can target multiple genes. Gene ontology (GO)-based clustering analysis revealed that the majority of the endogenous target genes were represented in various categories of biological and metabolic processes, such as cell cycle, organelle organization, protein and lipid synthesis, cell signaling and catabolic processes, which are important processes required for the growth and development of fungi (Figure 3).

**Figure 4:**
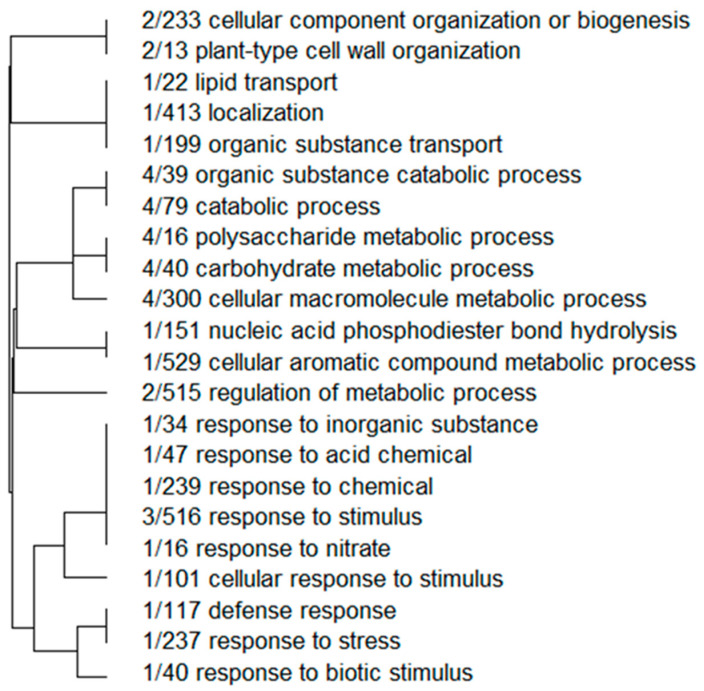
Predicted hop target clustering of selected hop transcripts by their biological process according to enriched gene ontologies; the values represent the number of selected milRNA targets in each enriched GO annotation.

Strikingly, the hop transcriptome contained 661 target mRNAs, and among them, 370 represented unique target sites of one of the nine validated *V. nonalfalfae* milRNAs, and the remaining 291 transcripts contained target sites for two or more milRNAs. The majority of the target transcripts were predicted to be targets of a single milRNA (conserved-miR-7044). The GO functional annotation, falling under the term biological processes, indicated that most of the predicted target transcripts belonged to plant defense responses, biotic and abiotic stress responses (Figure 4), metabolic process regulation, carbohydrate and polysaccharide metabolism and cell wall organization.

### 2.5. 5′ RLM-RACE Validation of the Predicted Target Gene Models

**Figure 5:**
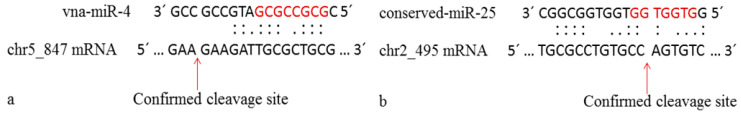
Schematic representation of the experimentally validated cleavage sites of the target mRNAs; (**a**)—evm.model.chr5_1271NY.847 and (**b**)—evm.model.chr2_1770NN.495; the seed region of mature milRNA is highlighted in red.

Experimental validation of the cleavage of the target transcripts was performed for four predicted target genes of *V. nonalfalfae*. MilRNA-associated cleavage of the target gene models using the modified 5′ RLM-RACE approach, comprising of RNA adaptor ligation, nested PCR, colony PCR and sequencing (Figure S5) was confirmed for two targets, the gene model evm.model.chr5_1271NY.847 (chr5_847), the component of the proteasome, and evm.model.chr2_1770NN.495 (chr2_495), a r3h domain-containing protein. However, in both gene models, the psRNATarget-predicted mRNA cleavage site was not detected in the sequences of the nested PCR products. In the case of chr5_847, which was predicted to be associated with vna-miR-4 and cleaved between nucleotides 5166 and 5167, the identified cleavage site resided 16 nt downstream of the predicted cleavage site, between nucleotides 5182 and 5183. This specific cleavage site was observed in 14 sequenced plasmids, transformed with the nested PCR product of the chr5_847 gene model. The identified cleavage site was screened with psRNATarget using less stringent parameters, and the corresponding sequence of the gene model cleavage site was successfully identified. The chr5_847 mRNA associated with vna-miR-4 was determined to be cleaved between 15 and 16 nt of the mature milRNA in the interaction (Figure 5a). 

Similar observations were encountered when investigating the interaction between the conserved-miR-7044 and chr2_495 mRNA, which is predicted to be cleaved at the 1890/1891 nt position. After screening the sequences of the amplified nested PCR product sequences, the cleavage site was determined to be 91 nt downstream of the predicted cleavage site. Using the psRNATarget search for additional predictions of cleavage sites within the gene model chr2_495, the experimentally identified cleavage site was detected, residing between nucleotides 1981 and 1982 of the gene model sequence. Cleavage occurred between the sixth and seventh nucleotides of the milRNA in the interaction. Both the experimentally validated cleavage sites and the associated milRNA interactions were schematically represented (Figure 5b), and, thus, confirmed the activity of RNAi and the associated milRNAs in *V. nonalfalfae*.

## 3. Discussion

Filamentous fungi comprise of many important plant and human pathogens, symbionts and economically important fungi. Their RNAi pathways have been intensively investigated in recent years [17,39]. RNAi is a conserved gene regulatory mechanism in the fungal kingdom, and only a few fungi have been reported to have lost the capability of silencing genes via RNAi; the mechanism is present in both ascomycete and basidiomycete fungi [16,39,40]. Initial studies of the RNAi in fungi included identification and functional characterization of the core components of RNAi; the RdRP polymerase transcribing the milRNAs, DCL enzyme processing the milRNA precursors and the AGO, the catalytic component of the RNAi. The core components of the RNAi have been identified and implemented in growth and development, as well as in the pathogenicity of several pathogenic plant fungal species, such as *Mucor circinelloides* [41,42,43], *Fusarium graminearum* [20] and *Magnaporte oryzae* [44], including *V. nonalfalfae* [11], the pathogen investigated in the present study. In addition, several published reports have found microRNA-like RNAs in filamentous fungi, representing important human and plant pathogenic species, symbionts and industrially important fungi [45,46,47,48,49,50], together with the first milRNAs reported in the model fungus *Neurospora crassa* [51]. Studies of novel milRNAs have been supported by the breakthrough in sequencing technologies, enabling the construction of deep transcriptome sequencing libraries and the detection of millions of sequencing reads using next-generation sequencing platforms. Sequencing data have been subsequently analyzed using bioinformatics tools developed hand in hand with sequencing technologies to ensure the processing of large generated datasets, most of which rely on RNA secondary structure analysis for the prediction of sRNA and miRNA regions in the investigated genomes [52]. For the identification of *V. nonalfalfae* milRNA, the common approach of sRNA sequencing using the NGS Ion Torrent platform and milRNA prediction with MIReNA software was used in the present study. 

For each of the eight sequencing data sets for the highly virulent T2 and less virulent Rec isolates, approximately 10 million sequencing sRNA reads were generated with an average length between 15 and 30 nt, coinciding with the length of fungal siRNAs, defined between 18 and 30 nt [12,16]. The MIReNA analysis results revealed the existence of milRNA structures in *V. nonalfalfae*, as milRNA precursors were predicted in all the datasets, whereas a total of 27 milRNA precursors had typical miRNA secondary structure and fulfilled several different criteria for true miRNA candidates [36]. The length of the predicted *V. nonalfalfae* milRNAs, which ranged from 18 to 29 nt, correlated with the reported length of fungal sRNAs and milRNAs, making the MIReNA algorithm suitable for predicting fungal milRNAs due to its flexibility in identifying miRNAs with non-characteristic features [39,53]. 

Experimental validation of miRNAs is usually based on the detection of short miRNA sequences using modified RT-qPCR, which is defined as the gold standard for miRNA validation due to its sensitivity and specificity towards short RNA sequences [54]. The expression of the nine identified and validated *V. nonalfalfae* milRNAs (Table 2) was confirmed with high confidence using the modified stem-loop RT-qPCR method [37], in which Sybr Green chemistry and primers covering the entire milRNA sequence were used to assure the specificity of the amplification of each mature milRNA sequence. Of the nine validated milRNAs, eight were novel, with no match in the miRBase database, and were designated “vna-miR” in accordance with the miRNA nomenclature. One fungal milRNA, named “conserved-miR-7044” aligned perfectly with the mouse mmu-miR-7044. However, we cannot speculate on the role of this miRNA for the mouse nor the fungi, as the targets of the mouse miRNA were not searched or homologoues target genes identified. The BLASTX analysis revealed three *V. nonalfalfae* milRNAs (vna-miR-3, vna-miR-4 and vna-miR7) residing within coding or exonic regions of the annotated *V. nonalfalfae* gene models [10]. In *Coprinopsis cinereia*, Lau et al. (2018) annotated two exonic fungal milRNAs, a phenomenon also observed in the animal kingdom, in which exonic miRNAs were discovered originating from untranslated mRNA transcripts [55,56]. According to the RNA-Seq data (available in our laboratory) of *V. nonalfalfae*, the predicted target gene models, encoding the exonic milRNAs, are not expressed. The overall number of predicted and validated *V. nonalfalfae* milRNAs correlates with discoveries in other fungal species, where up to 20 milRNAs have been experimentally confirmed, mainly using the RT-qPCR approach [18,19,20,21,22,46,48,57,58].

Targets were predicted for all nine identified and validated *V. nonalfalfae* milRNAs in the fungal genome as well as in the hop transcriptome. Further fungal target validation using the 5′ RLM-RACE method confirmed the activity of the milRNA-mediated RNAi in *V. nonalfalfae*, as for two predicted target fungal gene models, target mRNA cleavage was detected within the milRNA-target recognition site. However, the efficiency of the 5′ RLM-RACE method proved to be relatively low since only two alternative cleavage sites were detected and confirmed out of the four tested in the RACE experiment. To achieve a better output, the PARE method of ligating RNA transcripts and subsequently performing high-throughput sequencing [59] could be used as an alternative, enabling the screening of a much high number of miRNA-mRNA target cleavage sites. 

In *V. dahliae*, a close relative of *V. nonalfalfae*, one out of seven predicted milRNAs, VdmilR1, was detected and confirmed using the RNA gel blotting approach. The confirmed *V. dahliae* milRNA was implicated in the repression of a virulence gene, thus affecting the pathogenicity of the fungus, which has been confirmed in target knock-out experiments [24]. To investigate the potential role of the identified and validated *V. nonalfalfae* milRNAs for the pathogenicity of the fungus, pathotype-specific and tissue-specific expression analysis was conducted, implementing the absolute quantification approach. The highest copy numbers of all nine *V. nonalfalfae* milRNAs were found in the mycelia of the highly virulent isolate T2 grown in xylem-simulating media that simulates the environment of the host plant (Figure 2), in which the fungus actively spreads and blocks the vasculature tissue of the host [3,60]. Comparing the expression of the investigated milRNAs between T2_XSM and Rec_XSM, a significantly higher expression of the milRNAs was observed in the highly virulent T2 isolate. We, therefore, propose a model of intense RNAi activity in the highly virulent *V. nonalfalfae* pathotypes. The hypothesis was also supported by observations in our previous study, where the core components of RNAi were identified and demonstrated to be upregulated in the mycelia XSM of highly virulent T2 isolate, when fungi are actively spreading in host hop plants [11]. On the other hand, in the less virulent Rec isolate, the expression of the *V. nonalfalfae* milRNAs suggested the involvement of RNAi in the regulation of endogenous genes during sporulation, as all the milRNAs were upregulated in the conidia of the fungus, with significantly higher milRNA copy numbers observed compared to the mycelia grown in XSM (Figure 2). The critical role of RNAi as a sporulation factor was also observed in other fungal species, in which milRNAs were associated with the regulation of specific developmental stages in *Trichophyton rubrum* [61] and *Metarhizium anisopliae* [62].

RNAi and associated milRNAs are known to mediate the virulence of fungal pathogens by regulating the endogenous expression of virulence genes [24] or toxin synthesis [63] and can even affect host defense responses via cross-kingdom RNAi and lead to the export of RNAi signals, as shown in *B. cinereia* and *Puccinia striformis* [31,33,64]. To elucidate the involvement of the *V. nonalfalfae* milRNAs in either the pathogenicity or development and sporulation of the fungus, the targets of the identified and validated milRNAs were searched and analyzed in the *V. nonalfalfae* genome and in the host (hop) transcriptome. Interestingly, in the hop transcriptome, significantly more targets were predicted, of which the majority represented transcripts involved in host defense pathways, suggesting a possible cross-kingdom RNAi movement of RNAi signals from *V. nonalfalfae* into hop host plants, similar to the case of *Rhizophagus irregularis*, for which a total of 237 plant targets of fungal milRNAs were predicted [65]. It was experimentally determined for the pathogenic fungus *B. cinerea* that the small RNA Bc-siR37 is exported into the host, where it affects the expression of at least three *A. thaliana* genes, resulting in increased disease susceptibility [33]. By exporting RNAi signals as well as milRNAs, *V. nonalfalfae* can potentially affect the defense mechanism in the host hop plant. However, further studies and experimental validation are needed, especially at the level of the extracellular vesicular transport of the sRNA and milRNA signals, which is assigned as the prime mode for the exchange of RNA signals between kingdoms and in host-pathogen interactions and pathosystems [64,66,67].

## 4. Materials and Methods

### 4.1. Verticillium nonalfalfae Culture Preparation

The two *V. nonalfalfae* isolates, the highly virulent hop pathotype PV1 (isolate designation—T2) and less virulent pathotype M (isolate designation—Rec), were obtained from the culture collection of the Slovenian Institute for Hop Research and Brewing, and stock cultures were further maintained on potato dextrose agar supplemented with Czapek Dox (CD) broth at 4 °C. The study included the four distinct morphological stages of these two isolates: (1) conidia, (2) mycelia grown in liquid CD media, (3) mycelia grown in liquid xylem-simulating media (XSM) [68] and (4) resting mycelia. For mycelia production, spores, harvested in sterilized water, were inoculated (100 µL) in liquid CD (Sigma Aldrich, St. Louis, MO, USA) broth and in liquid XSM media and the cultures were grown at 25 °C and 120 rpm for 6 days. The production of conidia was induced by growing cultures in Petri dishes containing CD broth solidified with 0.8% agar (Duchefa, Haarlem, The Netherlands) at room temperature in the dark. The conidia of both cultures were harvested after two weeks of growth. For resting mycelia production, both the isolates were grown in solid media containing plum extract (PLYA) [69] for two months at room temperature and in the dark. After collection, all samples were frozen in liquid nitrogen and stored at −80 °C for further analysis.

### 4.2. Small RNA Extraction and Ion Torrent NGS Sequencing

The small RNA fraction (<200 nt) was isolated from 100 mg of all four tissue samples for both fungal pathotypes using a mirVana™ miRNA Isolation Kit (Invitrogen™, Waltham, MA, USA), according to the manufacturer’s protocol. The concentration of the sRNAs was determined using a Qubit 2.0 (Thermo Fisher Scientific, Waltham, MA, USA) fluorimeter and a Qubit microRNA Assay Kit (Thermo Fisher Scientific, Waltham, MA, USA), following the manufacturer’s instructions. The quantity of the milRNA fraction was assessed using an Agilent Bioanalyser 2100 (Agilent, Santa Clara, CA, USA) and an Agilent Small RNA Kit (Agilent, Santa Clara, CA, USA). The small RNA sequencing library was constructed with an Ion Total RNA Seq Kit v2 (Ion Torrent™) using 3 µL of each isolated sRNA fraction. Each of the eight samples was tagged with different Ion Xpress™ Barcode Adapters (Ion Torrent™, Waltham, MA, USA), and the transcribed cDNA was pooled in two matrixes depending on the fungal pathotype (T2 and Rec). The pooled cDNA matrixes were amplified using the Ion OneTouch™ 2 System (Ion Torrent™, Waltham, MA, USA) and loaded onto two semiconductor sequencing chips. High-throughput sequencing was performed using an Ion Proton™ System (Ion Torrent™, Waltham, MA, USA).

### 4.3. Sequence Analysis and milRNA Prediction

The raw sequencing reads were trimmed to remove adaptor sequences, sample-specific barcodes and reads with ambiguous nucleotides using the Torrent Suite Software (Ion Torrent™, Waltham, MA, USA). The quality control analysis was performed for each of the eight data sets using the FastQC software package (version 0.11.2). Next, the sequenced reads were collapsed to obtain unique reads for each sRNA sample of the two isolates. The rRNA, tRNA, snRNA and snoRNA species were identified using the Infernal software [70] against the Rfam database (version 14.7) with default parameters.

The prediction of potential milRNAs was performed using the MIReNA software [35], which aligns sequencing reads to the reference genome and identifies candidate precursors that have a hairpin secondary structure. The search was conducted for all eight collapsed sequence data sets representing the four types of fungal material for the highly virulent isolate T2 and the less virulent isolate Rec and for the combined data sets for all the four Rec and all the four T2 sequences (Rec_all and T2_all, respectively). The highly virulent *V. nonalfalfae* isolate T2 [10], scaffolded to the optical map (data unpublished), was used as the reference genome, and the algorithm was run with the default parameters. Furthermore, the hairpin structures of the predicted milRNA precursors were analyzed using the mfold online tool (http://www.unafold.org/mfold/applications/rna-folding-form.php, accessed on 8 March 2021), and were manually inspected for the appropriate hairpin secondary structure. The potential *V. nonalfalfae* milRNA precursors were further categorized and selected based on the previously proposed criteria [36]. The screened milRNA precursor candidates were further aligned with the miRBase data base (release 22.1, http://www.mirbase.org/, accessed on 12 March 2021) to determine the possible conservation of the milRNA sequences with known miRNAs. In the final step, local BLASTX analysis (version 2.11.0) was performed of the milRNA candidates against the NCBI database of protein sequences to scan their possible origins from the coding sequences.

### 4.4. MilRNA Validation and Expression Analysis

Validation and quantification of the selected milRNA candidates were performed using the stem-loop RT-qPCR method as described previously [37]. The milRNA-specific stem-loop primers were designed and reverse transcribed using 10 ng of RNA with TaqMan™ MicroRNA Reverse (Applied Biosystems™, Waltham, MA, USA), according to the manufacturer’s protocols. The cDNA was serially diluted 4-fold, and each reverse transcribed milRNA was amplified via qPCR using the Fast SYBR™ Green Master Mix (Applied Biosystems™, Waltham, MA, USA) and 1.5 µM milRNA-specific forward primer, 0.7 µM universal reverse primer and 1 µL of diluted cDNA in three biological replicates. The temperature program for milRNA amplification was 10 min at 95 °C, followed by 40 cycles of 95 °C for 10 s and 60 °C for 30 s, and each milRNA was tested in three biological and three technical replicates. The milRNA-specific forward qPCR primers were developed based on the instructions in the Kramer protocol [37]; however, wherever possible, longer primers covering the entire milRNA sequence were designed and used in qPCR to ensure the specificity of amplification.

In several cases, for which the milRNA-specific qPCR primers did not cover the entire length of the mature milRNA, the milRNAs were amplified via PCR using a KAPA HiFi HotStart PCR Kit (Roche, Basel, Switzerland), according to the manufacturer’s protocol, with each 25 µL reaction containing 3 µL of synthesized cDNA. The PCR products were size separated and visualized via 2.5% agarose gel electrophoresis, and amplicons of expected length (approximately 70 bp), representing mature milRNA and the added universal stem-loop sequences, were excised from the gel. The PCR products were cleaned using a Silica Bead DNA Gel Extraction kit (Thermo Fisher Scientific, Waltham, MA, USA) and dissolved in 10 µL of nuclease-free water. Five microliters of the blunt-end PCR fragments was cloned into the pJET1.2/blunt plasmid vector, in accordance with the protocol of the CloneJET PCR Cloning Kit (Thermo Fisher Scientific, Waltham, MA, USA). The plasmid vectors were transformed into competent *Escherichia coli* bacterial cells, strain DH5α, and positive clones were grown overnight in liquid LB media supplemented with carbenicillin (25 g/L LB broth high salt (Duchefa, Haarlem, The Netherlands), 100 mg/L carbenicillin (Sigma Aldrich, St. Louis, MO, USA)) at 37 °C and 120 rpm. Plasmid DNA was isolated using a High Pure Plasmid Isolation Kit (Roche), and the plasmid vectors were sequenced using a BigDye™ Terminator v3.1 Cycle Sequencing Kit (Applied Biosistems™, Waltham, MA, USA). The sequencing reactions were cleaned using the ethanol precipitation method and analyzed on an ABI 3130XL sequencer (Applied Biosistems™, Waltham, MA, USA). The sequences were manually inspected for the presence of the mature milRNA sequence using the licensed CodonCode Aligner software (CodonCode Corporation, Centerville, MA, USA). 

The absolute quantification method for determining the milRNA copy number was used for quantifying milRNAs in specific tissues and in each of the two investigated pathotypes. The miRNA quantification standard miR-H1, described in the Kramer protocol [37], was used for qPCR amplification and quantification using the standard curve approach to calculate the milRNA copy number. Briefly, the miRNA quantification standard, synthesized by Invitrogen, was diluted to a concentration of 0.5 µM, and a series of 10-fold dilutions was prepared according to the instructions in the Kramer protocol. In the reverse transcription reaction, 2.5 µL of each miR-H1 dilution was used along with the specific stem-loop primer [37]. The cDNA of each dilution was amplified via qPCR using the Fast SYBR™ Green Master Mix replicated with three biological samples. The *V. nonalfalfae* milRNAs were reverse transcribed from RNA from the two fungal pathotypes isolated from conidia and mycelia grown in XSM and amplified via qPCR as mentioned above. The milRNA copy number was calculated from the standard curve equation and normalized based on the expression of the *V. nonalfalfae* reference genes Vna8.801 (splicing factor 3a2) and VnaUn.148 (DNA topoisomerase) as designed by Marton et al. [71]. Subsequently, the milRNA copy number was normalized per 1 ng of the RNA. For statistical analysis, we used the log10 transformation of the copy number (Count). Statistical analysis of the log10 (Count) for each milRNA in four different pathotype-tissue combinations (PTCs) was performed in two steps. In the first step, one-way analysis of variance (ANOVA) was performed for 36 combinations of PTC and milRNA. Second, for each milRNA, four comparisons were analyzed as the difference of the mean values of log10 (Count): (a) for Rec, XSM was compared with conidia; (b) for T2, XSM was compared with conidia; (c) for conidia, T2 was compared with Rec; (d) for XSM, T2 was compared with Rec. For each of these 36 comparisons (four comparisons for each of the nine milRNAs), the confidence interval (CI) was calculated, and inverse transformation was applied, and CI was obtained for the ratio of the mean values of Count. The R-Software version 3.2.6 was used with two additional packages, “ggplot2” for the plots and “multcomp” to assess the comparisons under study [72].

### 4.5. MilRNA Target Prediction and GO Analysis

We used psRNATarget (2017 update, https://www.zhaolab.org/psRNATarget/, accessed on 20 May 2021), an open-source algorithm for predicting miRNA targets, to determine the endogenous fungal target gene models in the annotated fungal genome and in the hop transcriptome (http://hopbase.cgrb.oregonstate.edu/, accessed on 20 May 2021) to elucidate possible trans-kingdom RNAi pathways in the *V. nonalfalfae*-hop interactions. The following prediction criteria were used: (1) expectation = 3; (2) no. of mismatches allowed in the seed region = 2; (3) HSP size = 18; and (4) UPE = 25; (5) translation inhibition range = 9–11. Subsequently, for both sets of predicted target transcripts, gene ontology-based annotation was performed by mapping them against the EggNOG database (http://eggnog45.embl.de/ (accessed on 26 May 201), version 4.5.1) using the “eggNOG-mapper v1” plugin (http://eggnog-mapper.embl.de/ (accessed on 26 May 2021), version 2.1.6). The “DIAMOND” mapping protocol with default parameter settings was used for the annotations. The GO annotation of the predicted target transcript was imported into R, and the open-source script for GO clustering, GO_MUW.R [73], was used to cluster the predicted target sites according to their GOs to determine their involvement in the main biological processes. 

### 4.6. Target Validation

The predicted endogenous target genes of *V. nonalfalfae* were validated with the 5′ RLM-RACE method for detection of miRNA target cleavage sites. We manually inspected the predicted endogenous target genes and selected eight genes, based on 2 mismatches between the milRNA seed region and target site for further analysis. The expression pattern of each selected gene was determined by analyzing the RNA-seq data available in JBrowser (http://88.200.30.139/browser, accessed on 15 June 2021) of the annotated *V. nonalfalfae* genome and RT-PCR. For RT-PCR analysis, 1 µg of total RNA isolated from different tissues from the two pathotypes was reverse transcribed into cDNA using a High-Capacity cDNA reverse Transcription Kit (Invitrogen™, Waltham, MA, USA). The gene-specific forward and reverse primers were designed with the Primer3 online tool (http://bioinfo.ut.ee/primer3-0.4.0/, accessed on 15 June 2021) and used for cDNA amplification with a KAPA HiFi HotStart PCR Kit (Roche, Basel, Switzerland), according to the manufacturer’s protocol. The products were analyzed on a 1% agarose gel, and the amplified genes were selected for ligation reactions. 

For 5′ RLM-RACE analysis, a FirstChoice™ RLM-RACE Kit (Invitrogen™) was used with some modifications, without alkaline phosphatase and acid pyrophosphatase treatment of the isolated RNA. First, 1000 ng of RNA was ligated with the 5′ RACE adaptor. The ligated RNA was reverse transcribed into cDNA and used in nested PCR with the outer and inner nested primers (Table S4) specifically designed for each target gene, according to the manufacturer’s protocol. The nested PCR products were analyzed on a 2% agarose gel, and the inner PCR fragments of the expected lengths were excised and cleaned with a Silica Bead DNA Gel Extraction Kit (Thermo Fisher Scientific™, Waltham, MA, USA). The cleaned inner PCR fragments were cloned into the pGEM^®^-T Easy Vector (Promega, Madison, WI, USA) and transformed into competent bacterial cells, according to the manufacturer’s instructions. The cleavage site of each gene was analyzed, and 12 bacterial colonies were used for recombinant plasmid DNA sequencing using a BigDye™ Terminator v3.1 Cycle Sequencing Kit (Applied Biosistems™, Waltham, MA, USA) and sequence analysis in the CodonCode Aligner, where specific cleavage sites were searched.

## 5. Conclusions

The wilting disease of hops, caused by the less virulent and most importantly, highly virulent isolates of the soil born fungus *Verticillium nonalfalfae*, is a major concern for hop growing regions in Europe and worldwide. The understanding of the *V. nonalfalfae*-hops pathosystem is crucial for the development of novel strategies for plant defense and disease management. In this perspective, the presented study gives an important information of the fungal pathogen, as for the other pathogenic plant fungi the RNAi and sRNA signals were implicated as one of the major virulence factors significantly contributing to the pathogenicity of the fungi. In our study, we reported, for the first time, on the milRNAs of the *V. nonalfalfae*. In total, 9 milRNAs were identified and confirmed to be expressed in both fungal pathotypes (Rec and T2, respectively). With the RLM-RACE method the milRNA-mediated target cleavage was observed, thus confirming the activity of the RNAi, guided by the fungal milRNAs. Moreover, with the qPCR expression analysis of the identified milRNAs differences in expression were observed between less virulent isolate Rec and highly virulent isolate T2, suggesting an important role of the milRNA-mediated RNAi for the virulence of the fungus. The later was additionally observed when targets of the *V. nonalfalfae* milRNAs were predicted in hop transcriptome, of which significant proportion was implicated in plant response to biotic stimulus and stress, indicating a potential cross-kingdom RNAi signaling and silencing of hop target genes. 

## Figures and Tables

**Table 1:** Summary of the sequencing and distribution of sRNAs among different *V. nonalfalfae* samples.

Sample	No. of All Total Sequenced Reads	No. of Unique Reads	Percent of Unique Reads [%]	No. of All rRNA/tRNA/sn-snoRNA	Percent of rRNA/tRNA/sn-snoRNA [%]
Rec_XSM	9,700,277	2,526,947	26	499,837	5.2
Rec_CD	10,360,750	2,322,612	22	542,831	5.2
Rec_conidia	12,804,008	2,090,161	16	684,176	5.3
Rec_resting	8,359,143	1,161,372	14	258,592	3.1
T2_XSM	8,595,353	1,510,766	18	562,534	6.5
T2_CD	4,387,811	912,222	21	256,280	5.8
T2_conidia	10,468,293	1,572,551	15	665,576	6.4
T2_resting	9,055,417	683,765	8	271,404	3.0

**Table 2:** Identified and confirmed *V. nonalfalfae* milRNAs with the characteristics of the precursor sequence.

Name	Length [nt]	Sequence (5′-3′)	Precursor Location	Precursor Length [nt]	MFE [kcal mol^−1^]	In Protein Coding Region?
vna-miR-1-5p	23	AAACGCTGATCTCCAAGTTACCT	Vna.chr3:112750..112859	110	−33.3	NO
vna-miR-2-5p	22	ACGGGTCCGGATATGCAGCTTG	Vna.chr4:1556903..1556813	101	−44.3	NO
vna-miR-3-5p	18	TGGCGCTGAGGGTGTCGA	Vna.chr1:2345083..2345190	108	−40.2	YES
vna-miR-4-5p	18	CGCGCCGCGATGCCGCCG	Vna.chr3:662853..662940	88	−43.8	YES
vna-miR-5-3p	21	TCAAGCCTGGGGCTCGCTTTG	Vna.chr4:3996299..3996386	88	−41.2	NO
vna-miR-6-5p	18	GGTCGACCTCTGGGCGCT	Vna.chr4:3834130..3834239	110	−50.6	NO
vna-miR-7-3p	18	TCCGGGATGAGGAGGAGC	Vna.chr4:933646..933736	91	−40.7	YES
mmu-miR-7044-5p (conserved-miR-7044)	18	GGTGGTGGTGGTGGCGGC	Vna.chr3:3285001..3285047	110	−61.0	NO
vna-miR-8-5p	20	TAGGTGGCTCTGAGGCATGG	Vna.chr-un:2863075..2863184	110	−39.8	NO

## Data Availability

The datasets generated and analysed during the current study are available in the NCBI Sequence Read Archive (SRA) repository (https://www.ncbi.nlm.nih.gov/sra/, accessed on 17 November 2021) under the BioProject accession number PRJNA624041. The lists of target mRNA transcripts of discovered milRNAs are openly available in FigShare repository at https://doi.org/10.6084/m9.figshare.11847177.v1 (accessed on 17 November 2021).

## Supplementary Materials

The following supporting information can be downloaded at: https://www.mdpi.com/article/10.3390/ijms23020900/s1.
