# Supplementary material for: Discovery of microRNA-like Small RNAs in Pathogenic Plant Fungus Verticillium nonalfalfae Using High-Throughput Sequencing and qPCR and RLM-RACE Validation
Source: Int J Mol Sci. 2022 Jan 14;23(2):900. doi: 10.3390/ijms23020900 (PMC8778906; doi:10.3390/ijms23020900)

Supplementary documents

Discovery of microRNA-like small RNAs in pathogenic plant fungus *Verticillium nonalfalfae* using high-throughput sequencing and qPCR and RLM-RACE validation

Taja Jeseničnik[1], Nataša Štajner[1], Sebastjan Radišek[2], Ajay Kumar Mishra[3], Katarina Košmelj[1], Urban Kunej[1] and Jernej Jakše[1*]

Agronomy Department[1], Biotechnical Faculty, University of Ljubljana, Ljubljana, Slovenia;

Slovenian Institute of Hop Research and Brewing[2], Žalec, Slovenia;

Biology Centre ASCR v.v.i, Institute of Plant Molecular Biology[3], České Budějovice, Czech Republic

Corresponding author: Jernej Jakše, PhD

Professor, Agronomy Department, Biotechnical Faculty, University of Ljubljana, Slovenia

Jamnikarjeva 101, Ljubljana, 1000, Slovenia

Phone: 00386 1 3203 280

Fax: 4231088

E-mail: Jernej.Jakse@bf.uni-lj.si

Supplementary Figure S1. Sequenced sRNA size plots for all eight samples, obtained with the QC analysis of the raw sequence data; A – Rec_XSM, B – Rec_CD, C – Rec_conidia, D – Rec_resting mycelia, E – T2_XSM, F – T2_CD, G – T2_conidia, H – T2_resting mycelia.

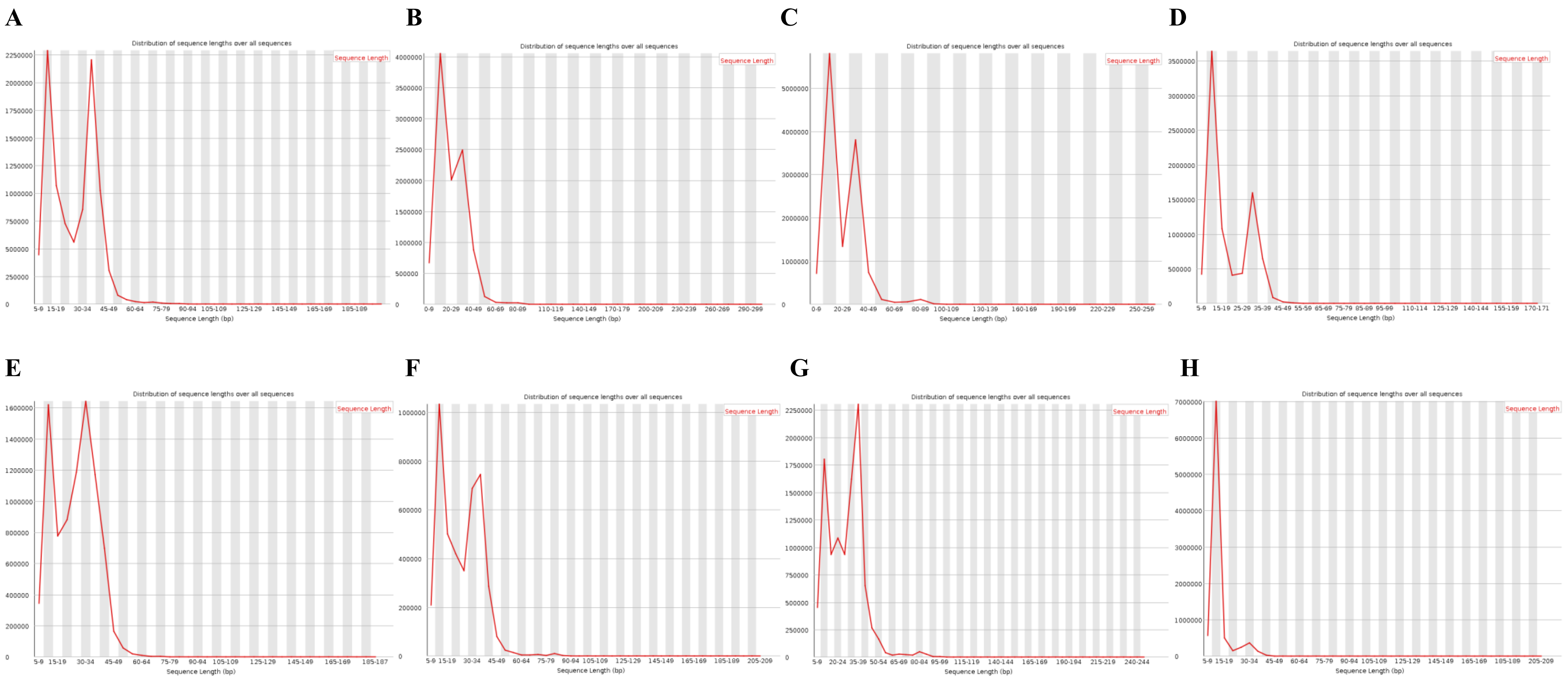

Supplementary Figure S2. Plots of proportional distribution of sRNAs for major classes of RNAs among samples obtained with comparison of clean reads with the Rfam database.

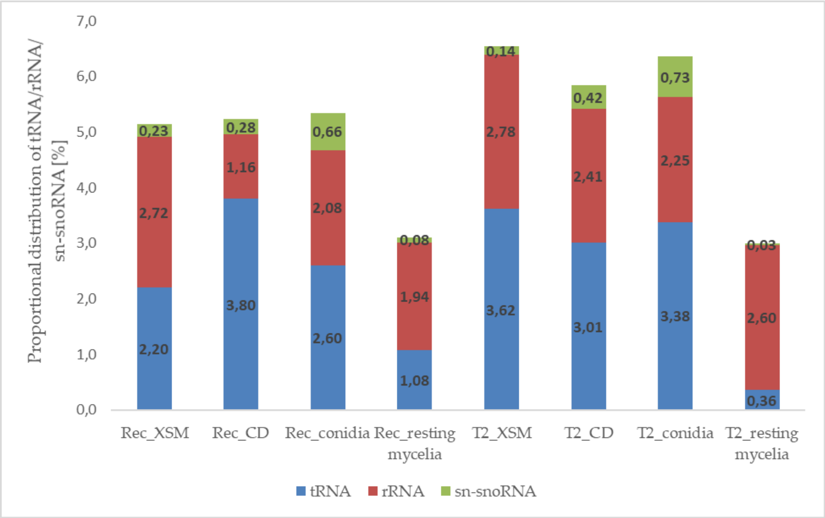

Supplementary Figure S3. The consensus sequences of cloned and sequenced milRNAs, for which the mature milRNA was not entirely covered by the qPCR primer. The mature milRNA sequence is highlighted in red and the mismatches of the obtained sequence compared to the mature milRNA in blue.

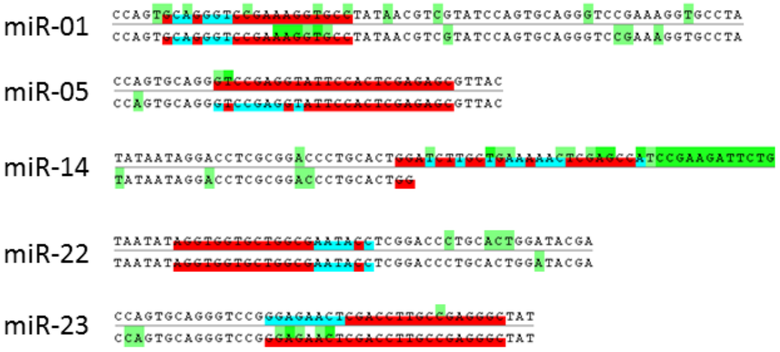

Supplementary Figure S4. The alignment of the *V. nonalfalfae* milRNA conserved-miR-7044 with the mouse miRNA mmu-miR-7044-5p, represented with the hit from the miRBase database.

Sequence search results

See the BLAST help pages for detailed information about the meaning of the scores shown here.

Accession	ID	Query start	Query end	Subject start	Subject end	Strand	Score	Evalue	Alignment
MIMAT0027992	mmu-miR-7044-5p	2	18	3	19	+	85	0.10	Align
MIMAT0027992	mmu-miR-7044-5p	1	15	5	19	+	66	3.9	Align
MIMAT0018899	tca-miR-3897-3p	2	17	2	17	+	62	8.3	Align
MIMAT0018899	tca-miR-3897-3p	1	16	4	19	+	62	8.3	Align
MIMAT0031418	mmu-miR-8112	2	17	4	19	-	62	8.3	Align

Alignment of Query to mature miRNAs

Query: 2-18   mmu-miR-7044-5p: 3-19   score: 85   evalue: 0.10

```
miR-25            2  guggugguggcggc  18
                     ||||||||||||||||
mmu-miR-7044-5p   3  guggugguggcggc  19
```

Supplementary Figure S5. The products of nested PCR amplification and colony PCR for investigated target mRNA cleavage in the 5' RLM-RACE analysis; A – the nested PCR products of the target evm.model.chr5_1271NY.847, amplified with two sets of primers; B – the products of the colony PCR for the two excised and cloned nested PCR fragments; C – the nested PCR products of the target evm.model.chr2_1770NN.495; D – the products of the colony PCR.

A

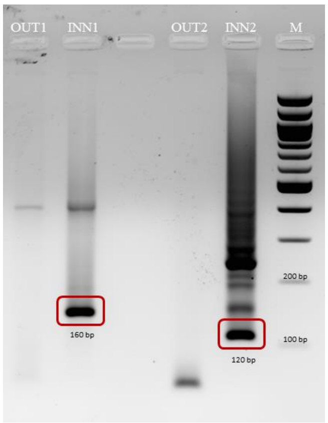

B

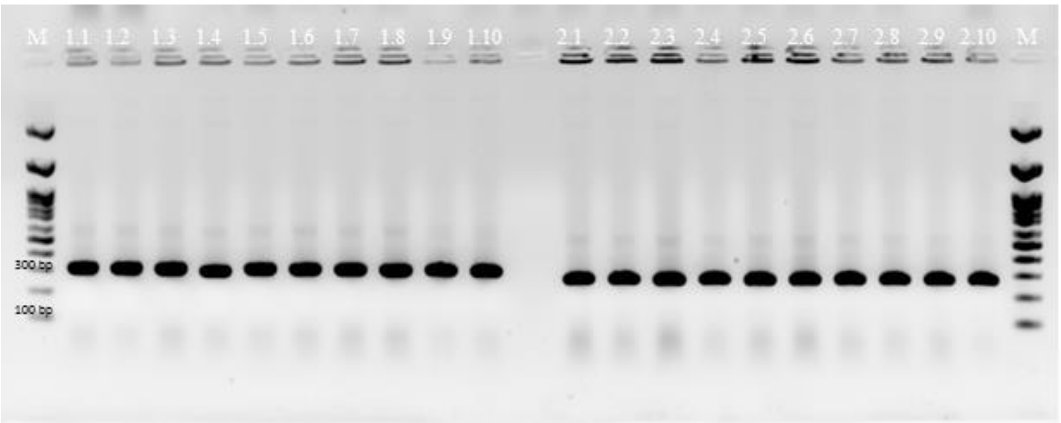

C

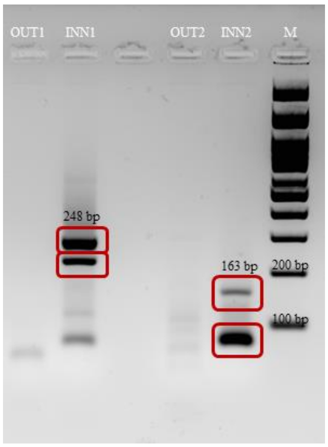

D

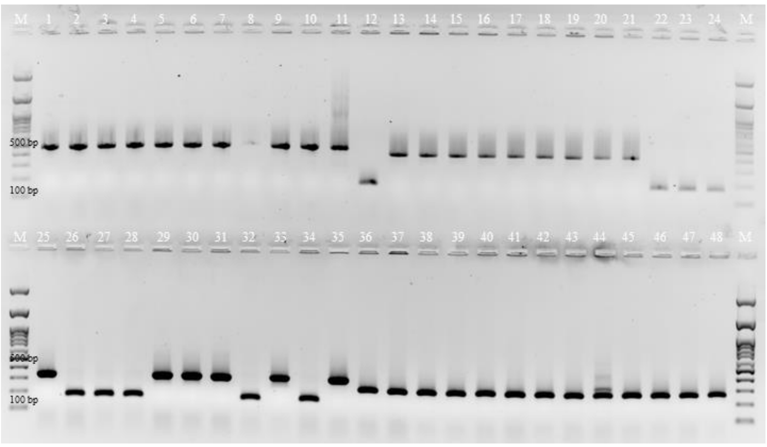

Supplementary Figure S6. The results of the statistical analysis for each PTC comparison for each identified *V. nonalfalfae* milRNA; the confidence intervals are shown on the original scale and represent the CI for the ratio of the corresponding Count means. The dashed horizontal line at 1 indicates whether the ratio deviates significantly from 1; this is generally true for T2: XSM vs conidia and for XSM: T2 vs Rec.

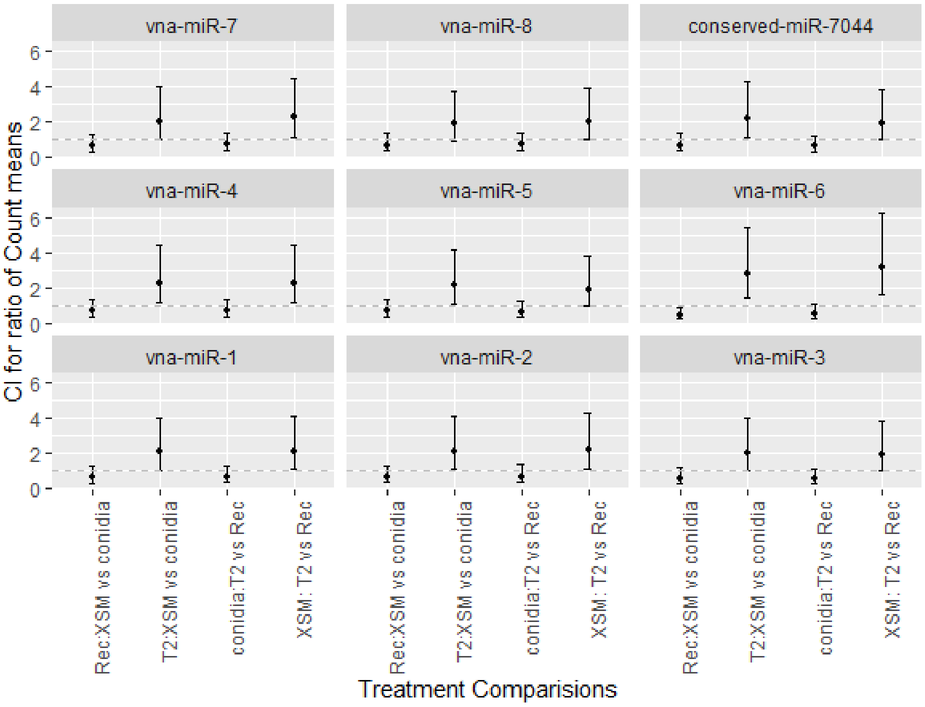

Supplementary Table S1. The list of all 27 selected true positive *V. nonalfalfae* milRNAs according to the precursor secondary structure and criteria for plant miRNA categorization (Mishra et al., 2015)

Sample name	milRNA name	Mature milRNA sequence (5'-3')	Mature milRNA length	Typical hairpin secondary structure	MFE	AMFE	MFEI	No. of mismatches between milRNA/milRNA*	In protein coding region
Rec_all	miR-01	G***GCACCT***TTCGGCGGCACC	19	YES	-40,2	-37,2	-0,6	5,5	YES
Rec_all	miR-02	A***AACGCT***GATCTCCAAGTTACCT	23	YES	-33,3	-30,3	-0,7	5,5	NO
Rec_all	miR-03	A***CGGGTCC***GGATATGCAGCTTG	22	YES	-44,3	-43,9	-0,8	5,5	NO
Rec_all	miR-04	T***CAAGCCT***GGGGCTCGCTTTG	21	YES	-41,2	-46,8	-0,9	5,5	NO
Rec_all	miR-05	G***CTCTCGA***GTGGAATGCGCGGAAA	24	YES	-41,6	-37,8	-0,6	5,5	YES
Rec_XSM	miR-06	G***ATGGCGG***CGTTGAGGGCGCA	21	YES	-40,3	-46,3	-0,7	8	YES
Rec_XSM	miR-07	T***GGCGCTG***AGGGTGTCGA	18	YES	-40,2	-37,2	-0,6	4,5	YES
Rec_XSM	miR-08	C***GCGCCGC***GATGCCGCCG	18	YES	-43,8	-49,8	-0,7	4	YES
Rec_XSM	miR-09	C***CAGCCGC***GTCGGTCCCGATGGGGAA	26	YES	-55,2	-51,1	-0,7	7,5	YES
Rec_XSM	miR-10	T***CAAGCCT***GGGGCTCGCTTTG	21	YES	-41,2	-46,8	-0,9	5,5	NO
Rec_XSM	miR-11	G***GTCGACC***TCTGGGCGCT	18	YES	-50,6	-46,0	-0,7	5	NO
Rec_XSM	miR-12	G***CTCTCGA***GTGGAATGCGCGGAAAGG	26	YES	-41,6	-37,8	-0,6	5	YES
Rec_CD	miR-13	G**GGTTGGC**GGTGATTTTGCGCAGTTTTCG	29	YES	-55,7	-50,6	-0,8	6	NO
Rec_CD	miR-14	G***GACCTCG***CGGCGACGGTCGACCT	24	YES	-46,4	-42,2	-0,6	8	YES
Rec_conidia	miR-15	C***GCCGCTT***CTGAGCAGAGTTCAAGTCTCACG	31	YES	-43,3	-37,3	-0,7	9	YES

Rec_conidia	miR-16	C***AGTCTGA***CAGCGTCCGGTTAGCTCGTG	28	YES	-37,3	-36,9	-0,7	8	YES
Rec_conidia	miR-17	A***GCAGTCT***CCCGGGCATTGCCT	22	YES	-49,5	-52,7	-0,8	6,5	YES
Rec_conidia	miR-18	T***TAGTGAA***TATGATAATGGTATATATGGT	29	YES	-24,4	-22,2	-0,9	9	YES
T2_all	miR-19	T***CTATCAT***GTCCATCTTCGCCGGTCGT	27	YES	-39,1	-40,3	-0,8	8,5	NO
T2_all	miR-20	A***GATTGTG***TAAAGGGGGTTATTCCAGG	27	YES	-29,5	-26,8	-0,5	7,5	NO
T2_XSM	miR-21	T***CCGGGAT***GAGGAGGAGC	18	YES	-40,7	-44,7	-0,7	6,5	YES
T2_XSM	miR-22	A***GGTGGTG***CTGGCGGCCGCG	20	YES	-50,8	-50,3	-0,7	6,5	YES
T2_conidia	miR-23	G***CCCTCGG***CAAGGTCGGCGGTCGC	24	YES	-52,0	-52,5	-0,7	5	YES
T2_conidia	miR-24	G***CGGGCCC***ACGTTGAGCT	18	YES	-40,2	-44,2	-0,6	4,5	YES
T2_conidia	miR-25	G***GTGGTGG***TGGTGGCGGC	18	YES	-61,0	-55,5	-0,7	3,5	NO
T2_conidia	miR-26	A***TACGGCG***ACAGCAGGTGGGTGGCATGA	28	YES	-57,0	-48,7	-0,8	8	NO
T2_conidia	miR-27	T***AGGTGGC***TCTGAGGCATGG	20	YES	-39,8	-36,2	-0,8	3,5	NO

Supplementary Table S2. List of designed and used stem-loop primers, specific for each selected candidate milRNA; the six milRNA specific nucleotides are marked in bold.

milRNA name	Name of the stem-loop primer	Primer sequence
miR-01	SL-miR-01	GTCGTATCCAGTGCAGGGTCCGAGGTATTCGCACTGGATACGAC**GGTGCC**
miR-02	SL-miR-02	GTCGTATCCAGTGCAGGGTCCGAGGTATTCGCACTGGATACGAC**AGGTAA**
miR-03	SL-miR-03	GTCGTATCCAGTGCAGGGTCCGAGGTATTCGCACTGGATACGAC**CAAGCT**
miR-04	SL-miR-04	GTCGTATCCAGTGCAGGGTCCGAGGTATTCGCACTGGATACGAC**CAAAGC**
miR-05	SL-miR-05	GTCGTATCCAGTGCAGGGTCCGAGGTATTCGCACTGGATACGAC**TTTCCG**
miR-06	SL-miR-06	GTCGTATCCAGTGCAGGGTCCGAGGTATTCGCACTGGATACGAC**TGCGCC**
miR-07	SL-miR-07	GTCGTATCCAGTGCAGGGTCCGAGGTATTCGCACTGGATACGAC**TCGACA**
miR-08	SL-miR-08	GTCGTATCCAGTGCAGGGTCCGAGGTATTCGCACTGGATACGAC**CGGCGG**
miR-09	SL-miR-09	GTCGTATCCAGTGCAGGGTCCGAGGTATTCGCACTGGATACGAC**TTCCCC**
miR-10	SL-miR-10	GTCGTATCCAGTGCAGGGTCCGAGGTATTCGCACTGGATACGAC**CAAAGC**
miR-11	SL-miR-11	GTCGTATCCAGTGCAGGGTCCGAGGTATTCGCACTGGATACGAC**AGCGCC**
miR-12	SL-miR-12	GTCGTATCCAGTGCAGGGTCCGAGGTATTCGCACTGGATACGAC**CCTTTC**
miR-13	SL-miR-13	GTCGTATCCAGTGCAGGGTCCGAGGTATTCGCACTGGATACGAC**CGAAAA**
miR-14	SL-miR-14	GTCGTATCCAGTGCAGGGTCCGAGGTATTCGCACTGGATACGAC**AGGTCG**
miR-15	SL-miR-15	GTCGTATCCAGTGCAGGGTCCGAGGTATTCGCACTGGATACGAC**CGTGAG**
miR-16	SL-miR-16	GTCGTATCCAGTGCAGGGTCCGAGGTATTCGCACTGGATACGAC**CACGAG**
miR-17	SL-miR-17	GTCGTATCCAGTGCAGGGTCCGAGGTATTCGCACTGGATACGAC**AGGCAA**
miR-18	SL-miR-18	GTCGTATCCAGTGCAGGGTCCGAGGTATTCGCACTGGATACGAC**ACCATA**
miR-19	SL-miR-19	GTCGTATCCAGTGCAGGGTCCGAGGTATTCGCACTGGATACGAC**ACGACC**
miR-20	SL-miR-20	GTCGTATCCAGTGCAGGGTCCGAGGTATTCGCACTGGATACGAC**CCTGGA**
miR-21	SL-miR-21	GTCGTATCCAGTGCAGGGTCCGAGGTATTCGCACTGGATACGAC**GCTCCT**
miR-22	SL-miR-22	GTCGTATCCAGTGCAGGGTCCGAGGTATTCGCACTGGATACGAC**CGCGGC**
miR-23	SL-miR-23	GTCGTATCCAGTGCAGGGTCCGAGGTATTCGCACTGGATACGAC**GCGACC**
miR-24	SL-miR-24	GTCGTATCCAGTGCAGGGTCCGAGGTATTCGCACTGGATACGAC**AGCTCA**
miR-25	SL-miR-25	GTCGTATCCAGTGCAGGGTCCGAGGTATTCGCACTGGATACGAC**GCCGCC**
miR-26	SL-miR-26	GTCGTATCCAGTGCAGGGTCCGAGGTATTCGCACTGGATACGAC**TCATGC**
miR-27	SL-miR-27	GTCGTATCCAGTGCAGGGTCCGAGGTATTCGCACTGGATACGAC**CCATGC**

Supplementary Table S3. The list of designed and used milRNA-specific qPCR primers

milRNA name	Name of the qPCR primer	Primer sequence	The length of mature milRNA covered by the primer	Amplicon length [bp]
miR-01	qP-miR-01	TTATAGGCACCTTTCGGCGGC	16	68
miR-02	LONG_qP-miR-02	CAGGAAACGCTGATCTCCAAGTTACCT	whole	71
miR-03	LONG_qP-miR-03	TTAACGGGTCCGGATATGCAGCTTG	whole	68
miR-04	LONG_qP-miR-04	TATAATTCAAGCCTGGGGCTCGCTTTG	28	71
miR-05	qP-miR-05	CGTAACGCTCTCGAGTGGAATGC	17	73
miR-06	qP-miR-06	AATATTGATGGCGGCGTTGAGGG	17	70
miR-07	qP-miR-07	CATATTATGGCGCTGAGGGTGTCGA	whole	69
miR-08	qP-miR-08	AATTATACGCGCCGCGATGCC	14	69
miR-09	qP-miR-09	TTATAACCAGCCGCGTCGGTC	15	75
miR-10	LONG_qP-miR-10	TATTATTCAAGCCTGGGGCTCGCTTTG	whole	71
miR-11	qP-miR-11	TTAATAGGTCGACCTCTGGGCGC	17	67
miR-12	qP-miR-12	GTTAATAGGTCGACCTCTGGGCG	16	76
miR-13	qP-miR-13	CCTCAAGGGTTGGCGGTGATTTT	17	78
miR-14	qP-miR-14	TATAATAGGACCTCGCGGCGACG	16	74
miR-15	qP-miR-15	TATCTTACGCCGCTTCTGAGCAGA	17	82
miR-16	qP-miR-16	TTCTGACAGTCTGACAGCGTCCG	17	77
miR-17	qP-miR-17	ACTATAAAGCAGTCTCCCGGGCAT	17	73
miR-18	qP-miR-18	GCCGCCGGTTAGTGAATATGATAAT	17	80
miR-19	qP-miR-19	CCGGGGCTCTATCATGTCCATCTT	17	77
miR-20	qP-miR-20	CGCGCTAGATTGTGTAAAGGGGG	17	76
miR-21	qP-miR-21	TTGATTCCGGGATGAGGAGGAGC	whole	66
miR-22	qP-miR-22	TAATATAGGTGGTGCTGGCGGC	16	69
miR-23	qP-miR-23	ATTATAGCCCTCGGCAAGGTCG	16	73
miR-24	qP-miR-24	TTAATAGCGGGCCCACGTTGAG	16	67
miR-25	qP-miR-25	TTATTAGGTGGTGGTGGTGGCG	16	67
miR-26	qP-miR-26	CTAAGAATACGGCGACAGCAGGT	17	77
miR-27	qP-miR-27	CTAATAGGTGGCTCTGAGGCATG	cela	68

Supplementary Table S4. The stem-loop RT-qPCR validation data obtained for each of the 9 confirmed *V. nonalfalfae* milRNAs. The obtained Ct values and calculated milRNA copy number are provided for both pathotypes and for the two tested conditions (mycelia grown in XSM and conidia).

milRNA	Average Ct value in Rec_XSM	Average Ct value in T2_XSM	Average Ct value in Rec_conidia	Average Ct value in T2_conidia	milRNA copy number in Rec_XSM (in 1 ng RNA)	milRNA copy number in T2_XSM (in 1 ng RNA)	milRNA copy number in Rec_conidia (in 1 ng RNA)	milRNA copy number in T2_conidia (per 1ng RNA)
vna-miR-1	25,6	25,4	25,0	25,3	1507254	4244356	7531453	1416864
vna-miR-2	17,3	16,9	16,8	16,9	1755614741	4209211143	10536393180	2010440158
vna-miR-3	16,0	15,9	15,1	15,8	1514616552	4920642809	8462471231	1255476477
vna-miR-4	30,9	30,5	30,4	30,6	199	510	1307	212
vna-miR-5	20,1	20,2	19,8	20,2	154180559	354454623	771226517	120033158
vna-miR-6	24,3	23,0	22,8	23,7	3906681	28101724	63925211	6109476
vna-miR-7	18,3	17,9	17,7	17,8	737196655	1849075547	4928196691	859362903
vna-miR-8	21,7	21,6	21,2	21,7	1541848764	3829139838	7294230200	2104388826
conserved-miR-7044	17,5	17,4	17,0	17,1	41442195	97506569	181588739	37344737

Supplementary Table S5. The list of designed “outer” and “inner” primers used in the nested PCR amplifications of the RNA adaptor ligated and reverse transcribed selected milRNA target gene models

Target name	Primer name	Primer sequence	Amplicon length [bp]
chr7_16	16_OUTER1	TCCGAGTGCAAAGACGTACA	329
	16_OUTER2	TGGTACTGGAAAGTGGCTGA	262
	16_INNER1	GACGTACCAATGGAGGCTGT	216
	16_INNER2	CTGTGAAAGCCGCTATTGAG	200
chr6_303	303_OUTER1	GCGGAACAAATACGTCTCGT	467
	303_OUTER2	CACGAGCGAGTAGCTGTCAA	320
	303_INNER1	GGTGCTCTTCTTCGCATCCT	189
	303_INNER2	CTCCAGCGGTGCTCTTCTTC	196
chr2_495	495_OUTER1	GATCTGAGGGCTGTTGTGGT	448
	495_OUTER2	ATCCAGGAGCACTGAACGAT	465
	495_INNER1	TTGGTGAGTAAGACGCAGGA	248
	495_INNER2	GTTGAACCCTTGCTGCCAAC	163
chr5_847	847_OUTER1	ACGCACTCGTACCAGACCTC	418
	847_OUTER2	GCAGCCTTATCATCTTGTCG	354
	847_INNER1	AATGTCGTCGAGGAGATCCA	160
	847_INNER2	GGCAAACTTCCACAGGTACG	124

Supplementary Figure S7. The secondary structures of identified and confirmed *V. nonalfalfae* milRNAs; a – vna-miR-1, b – vna-miR-2, c – vna-miR-3, d – vna-miR-4, e – vna-miR-5, f – vna-miR-6, g – vna-miR-7, h – vna-miR-8, I – conserved-miR-7044.

a

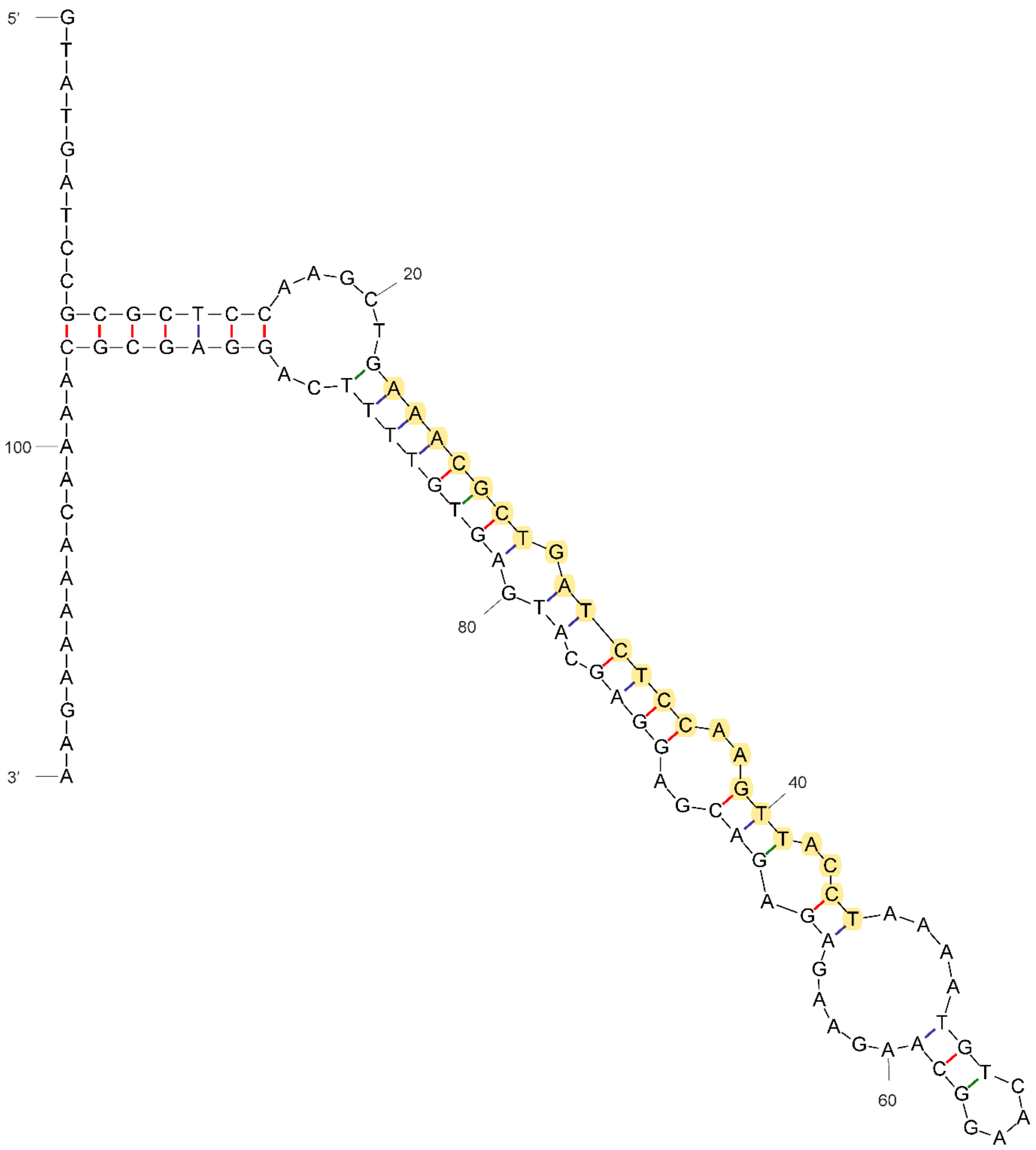

b

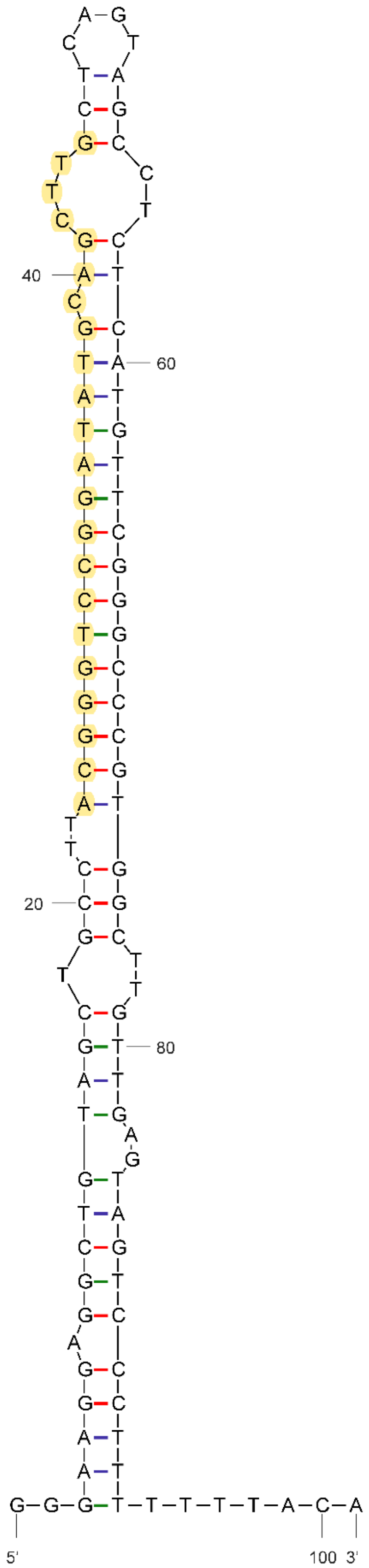

c

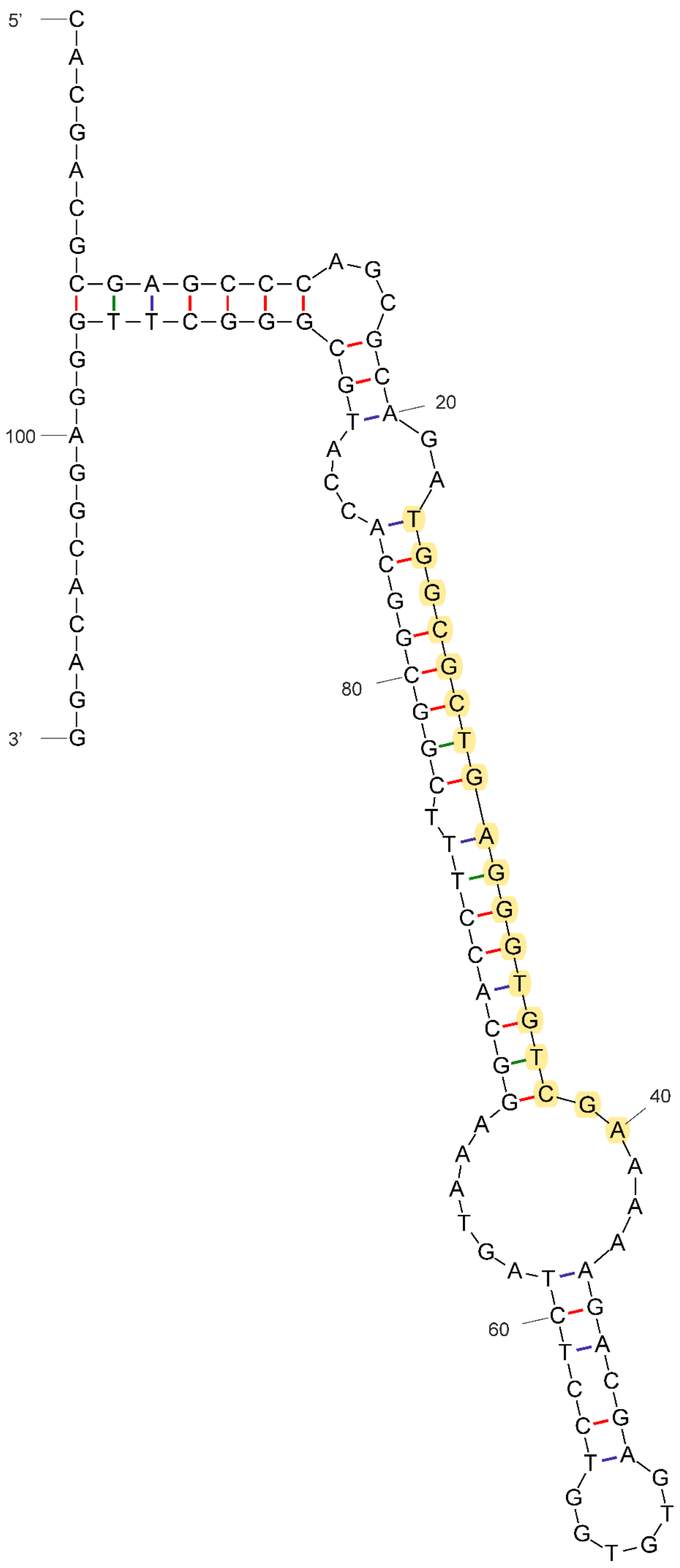

d

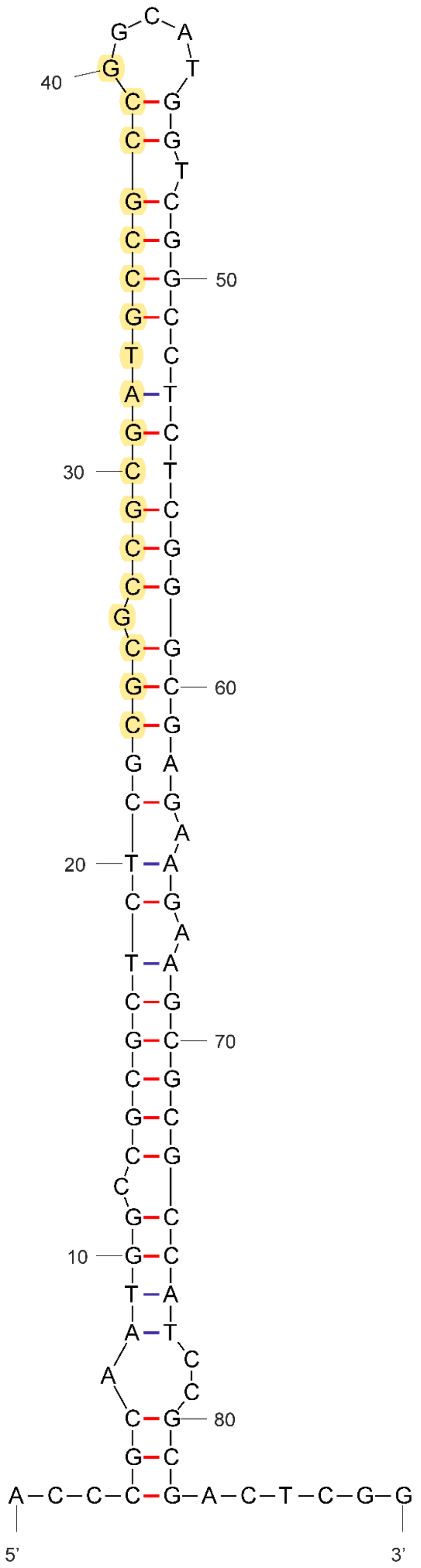

e

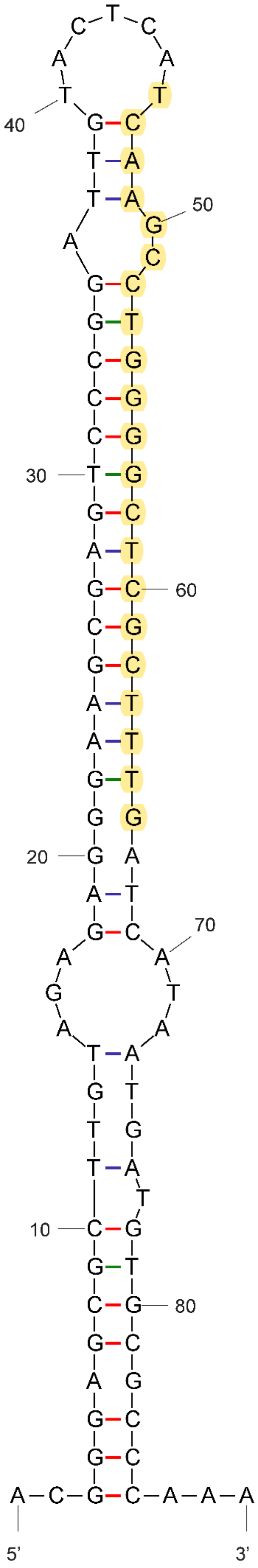

f

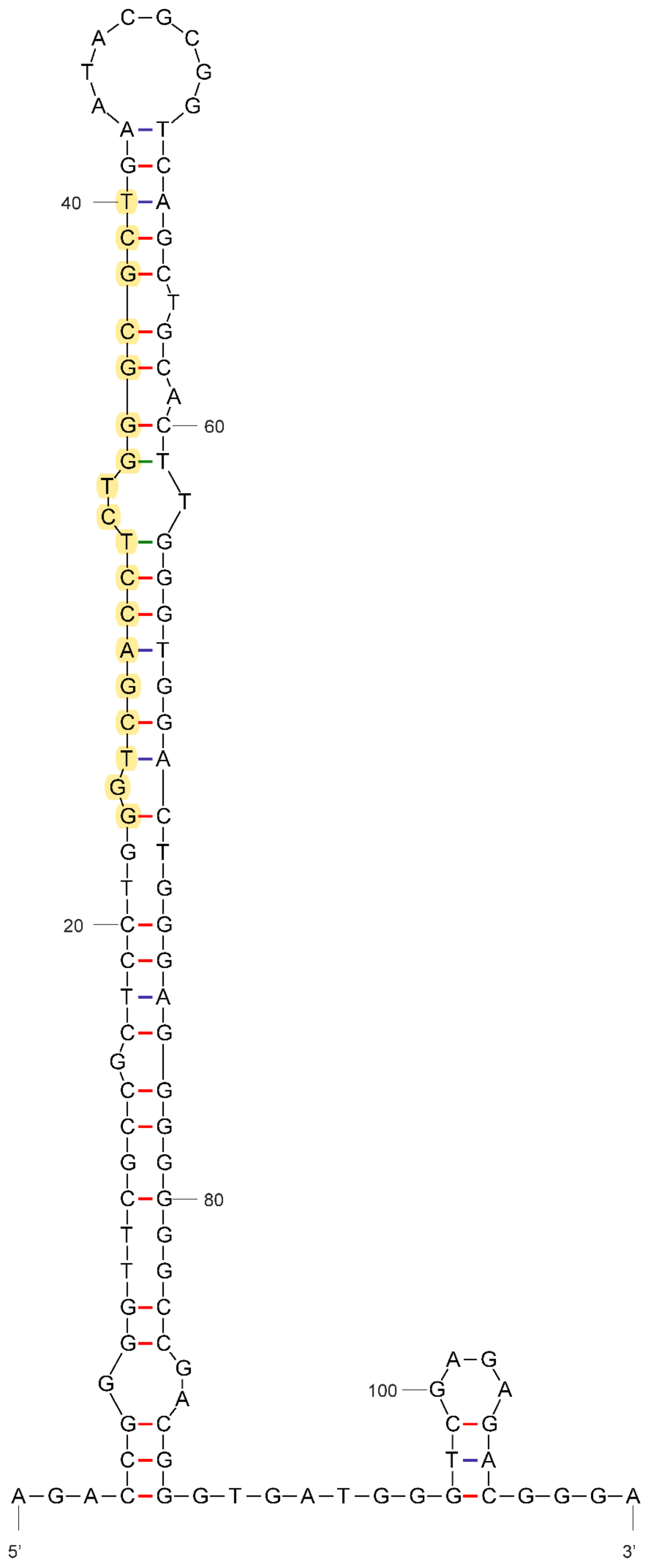

g

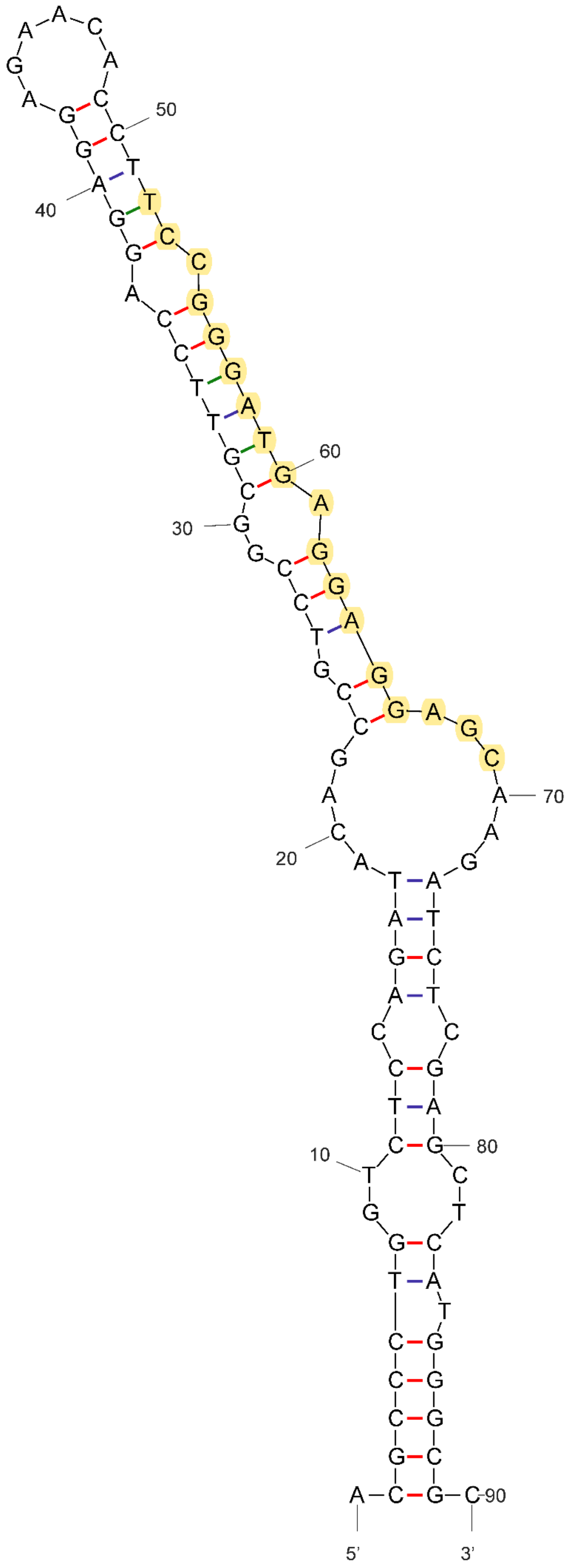

h

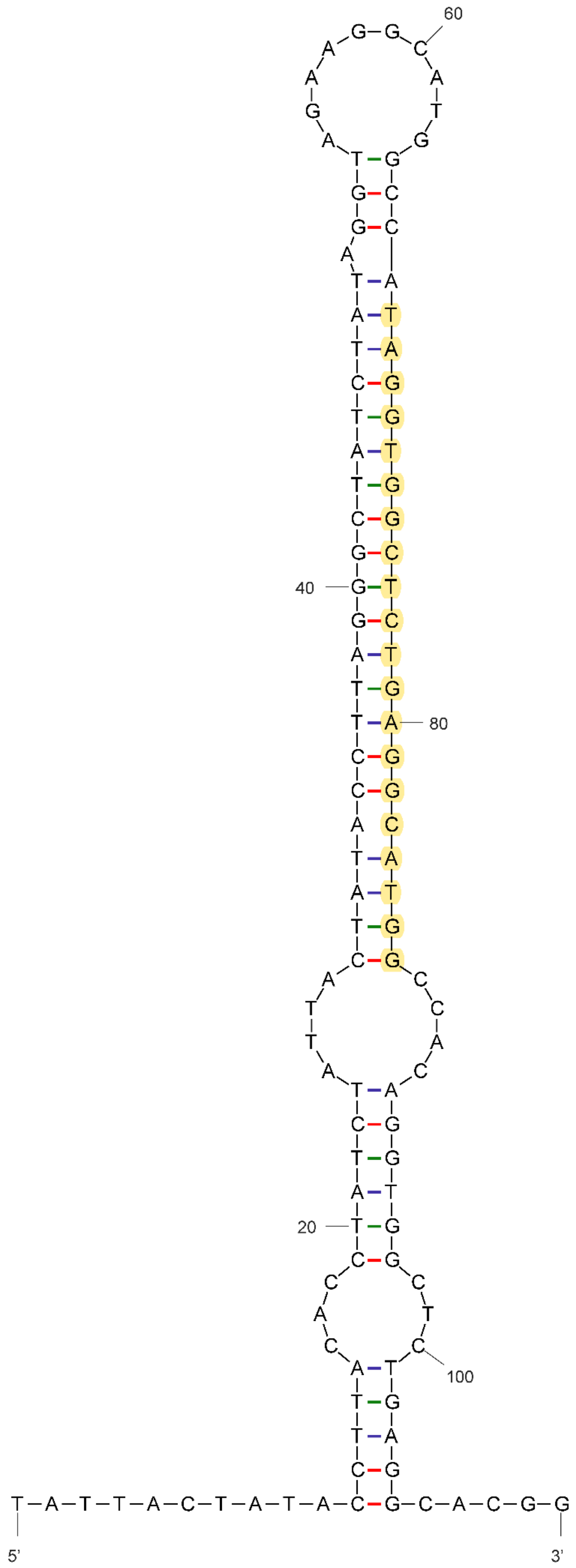

i

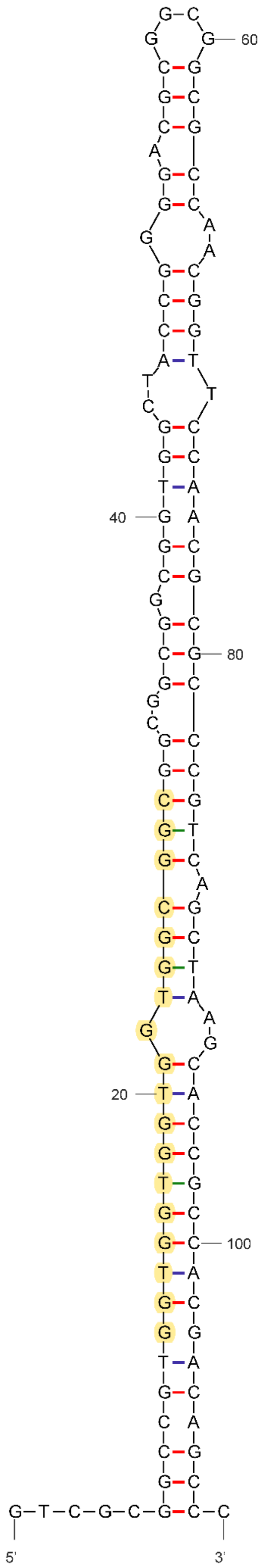

Supplement: Supplementary file 1 [file ijms-23-00900-s001.zip › ijms-1531327-supplementary.pdf]
